# An Injectable Zwitterionic Hydrogels with Multiple Intermolecular Interactions for Effective Prevention of Abdominal Adhesions

**DOI:** 10.1002/advs.202511757

**Published:** 2025-08-19

**Authors:** Na Wen, Yating Jiang, Yunhao Song, Jiachao Yang, Jinlin Long, Ying Wang, Xunbin Yu, Shiyun Lu, Tianhua Zhou, Xueping Huang

**Affiliations:** ^1^ College of Materials Science and Engineering Fuzhou University Fuzhou 350116 China; ^2^ State Key Laboratory of Structural Chemistry Fujian Institute of Research on the Structure of Matter Chinese Academy of Sciences Fujian 350002 P. R. China; ^3^ State Key Laboratory of Chemistry for NBC Hazards Protection, State Key Laboratory of Photocatalysis on Energy and Environment Fuzhou University Fuzhou 350108 China; ^4^ Fuzhou University Affiliated Provincial Hospital Fuzhou Fujian 350001 China; ^5^ Shengli Clinical Medical College Fujian Medical University Fuzhou Fujian 350001 China; ^6^ Department of Pathology Fujian Provincial Hospital Fuzhou Fujian 350001 China; ^7^ Department of Gastroenterology Fujian Provincial Hospital Fuzhou Fujian 350001 China

**Keywords:** antifouling, hydrogel, multifunctional, prevent abdominal adhesions, zwitterionic

## Abstract

Postoperative abdominal adhesions are the most common complication following abdominopelvic surgery, posing a significant burden on patients, clinicians, and society. However, current physical barriers often involve a tradeoff between preventing these adhesions and inhibiting inflammation. Herein, a one‐stone‐two‐birds strategy is presented to address this challenge through an injectable intertwined hydrogel containing sulfobetaine, modified aminocaproic acid (A6ACA), and ZnO nanoparticles (PSA‐ZnO hydrogel). This intertwined network is stabilized by multiple intermolecular coordination bonds, including hydrogen bonds and electrostatic interactions—enabling facile injection and resistance to abdominal creep stress. Experimental results demonstrate that PSA‐ZnO hydrogel fully reduce the severity of peritoneal adhesions in treated rats at both 7‐ and 14‐days post‐surgery, outperforming commercially available hyaluronic acid (HA) gel due to its superior antifouling, antibacterial (> 95% clearance of *E. coli* and *S. aureus*,), hemostatic (55 s), and wound healing properties (IL‐6 and TNF‐α decreased and VEGF increased). Unlike conventional barriers, PSA‐ZnO prevents foreign body formation by inhibiting blood clot organization and pathologic fibrin accumulation at wound sites. This integrated approach offers a clinically translatable solution for complete prevention of postoperative adhesions and inflammation, with the potential to improve patient outcomes and reduce healthcare burdens.

## Graphical abstract

An injectable PSA‐ZnO through multiple intermolecular including hydrogen bonds and electrostatic interactions for effectively prevents abdominal adhesions. The PSA‐ZnO hydrogel demonstrates superior wound healing promotion (≥ 95%) and more effective inhibition of scar formation compared to the commercial 3M dressing (≈80%), and prevents post‐operative abdominal adhesions superiorly compared to commercial anti‐adhesion HA gel.

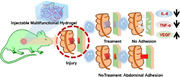

## 1 Introduction

Abdominal adhesions are a severe complication following abdominal or pelvic surgery, with more than 90% of patients developing adhesions.^[^ ^1^, ^2^, ^3^ ^]^ These adhesions can lead to female infertility, intestinal obstruction, and abdominal pain, significantly impacting patient health.^[^ ^4^, ^5^ ^]^ Current pharmacologic treatments, such as fibrinolytic agents (e.g., tranilast and N‐acetyl‐L‐cysteine),^[^ ^6^ ^]^ anti‐inflammatory drugs (e.g., ibuprofen and aspirin),^[^ ^7^ ^]^ and anticoagulants (e.g., heparin and hirudin),^[^ ^8^ ^]^ have some limitations, including a short residence time and rapid wound leakage.^[^ ^9^, ^10^ ^]^ Mechanical barrier materials, including natural polymer materials (e.g., Interceed, Adept, etc.) and synthetic materials (e.g., Seprafilm, Hyalobarrier, and SprayShield), have been approved by the Food and Drug Administration (FDA) or Conformite Europeenne (CE) for prevention, but they are difficult to apply and tend to adhere to everything from medical gloves to instruments, making surgery more difficult.^[^ ^11^, ^12^, ^13^ ^]^ Therefore, in order to improve the postoperative anti‐adhesion strategies, there is an urgent need to develop more direct and effective biological materials with easy operation (injection administration, etc.), sufficient wound site residence time, and superior anti‐adhesion effects.

Recently, hydrogel barrier materials have attracted considerable attention for the prevention of abdominal adhesions due to their advantage of injectability and better coverage of injured tissues.^[^ ^14^, ^15^ ^]^ Particularly those based on zwitterionic have gained attention for their injectability and superior coverage of injured tissues. For example, thiolated poly (SBMA‐coHDSMA) zwitterionic copolymers and sulfobetaine zwitterionic crosslinkers (BMSAB) are employed to create zwitterionic hydrogels by “click chemistry”. On days 7 and 14, all rats showed little adhesions, except for one rat with a score 1 adhesion.^[^ ^16^ ^]^ In another study, a zwitterionic polycarboxybetaine acrylamide (PCBAA) hydrogel was used to significantly reduce adhesion to abdominal fibroblast wall wounds within 4 days postoperatively, with a probability of successful adhesion prevention of more than 93% in 42 experimental animals.^[^ ^17^ ^]^ These hydrogels are extremely hydrophilic due to the presence of their anions and cations, which achieve better prevention of abdominal adhesions by allowing the zwitterionic polymers to form a dense hydrated layer on their surface, thereby preventing the deposition of fibrin.^[^ ^18^, ^19^, ^20^ ^]^ However, these hydrogels do not inhibit inflammation or regulate the balance between fibrin formation and lysis at the injury site, which is critical for adhesion prevention because peritoneal injury induces an inflammatory response. This limitation arises because zwitterionic polymers primarily provide a physical barrier through their hydration layer and antifouling properties but lack inherent bioactive functionalities necessary to modulate the inflammatory response and manage fibrin metabolism.^[^ ^21^, ^22^, ^23^, ^24^ ^]^ Another important factor in the prevention of abdominal adhesions is the hemostatic properties of hydrogels. Wound exudate, as well as clots and insoluble fibrin formed after hemorrhage coagulation, can cause excessive fibrin deposition and the formation of fibrin fibers. This further exacerbates the inflammatory response and promotes the development of abdominal adhesions.^[^ ^25^, ^26^ ^]^ To address these challenges, an effective strategy—such as incorporating anti‐inflammatory agents or bioactive components—is required to enhance the hydrogel's ability to regulate the inflammatory environment and maintain the balance between fibrin synthesis and degradation, thereby ensuring full prevention of postoperative abdominal adhesions.

While physiological fibrinolysis regulates blood clot formation, excessive fibrinolysis after trauma/surgery can cause coagulopathy, bleeding, and inflammation. Consequently, antifibrinolytic agents like FDA‐approved aminocaproic acid (A6ACA) are increasingly used to manage bleeding disorders stemming from hyperfibrinolysis. Simultaneously, ZnO nanoparticles – widely employed antibacterial agents – combat infection through Zn^2^⁺ release, reactive oxygen species generation, and electrostatic forces,^[^ ^27^, ^28^ ^]^ while also modulating inflammation and promoting healing. However, neither A6ACA nor ZnO addresses all key adhesion drivers: A6ACA risks promoting pathological fibrin persistence, while ZnO lacks intrinsic fibrin regulation. Furthermore, zwitterionic sulfobetaine.^[^ ^29^ ^]^ provides essential antifouling properties but cannot independently resolve bleeding or infection. This functional triad thus necessitates strategic integration, leveraging A6ACA's carboxyl‐Zn^2^⁺ electrostatic complexation and its amphiphilic structure‐mediated hydrogen bonding.^[^ ^30^, ^31^ ^]^ to create an intertwined hydrogel that simultaneously: (i) harnesses A6ACA's antifibrinolytic action while preventing pathological fibrin accumulation, (ii) exploits ZnO's antibacterial/anti‐inflammatory effects via controlled ion release, and (iii) utilizes sulfobetaine's antifouling capacity to prevent foreign body reactions – thereby establishing a multifunctional barrier that physically separates tissues while actively remodeling the wound microenvironment through orchestrated intermolecular interactions.

Thus, integrating aminocaproic acid, ZnO nanoparticles, and zwitterionic monomers such as sulfobetaine offers an effective strategy to develop intertwined hydrogels that function as adhesion barriers.^[^ ^31^ ^]^ This approach simultaneously provides physical separation and actively modulates the pathological wound healing microenvironment through intermolecular weak interactions.

**Scheme 1:**
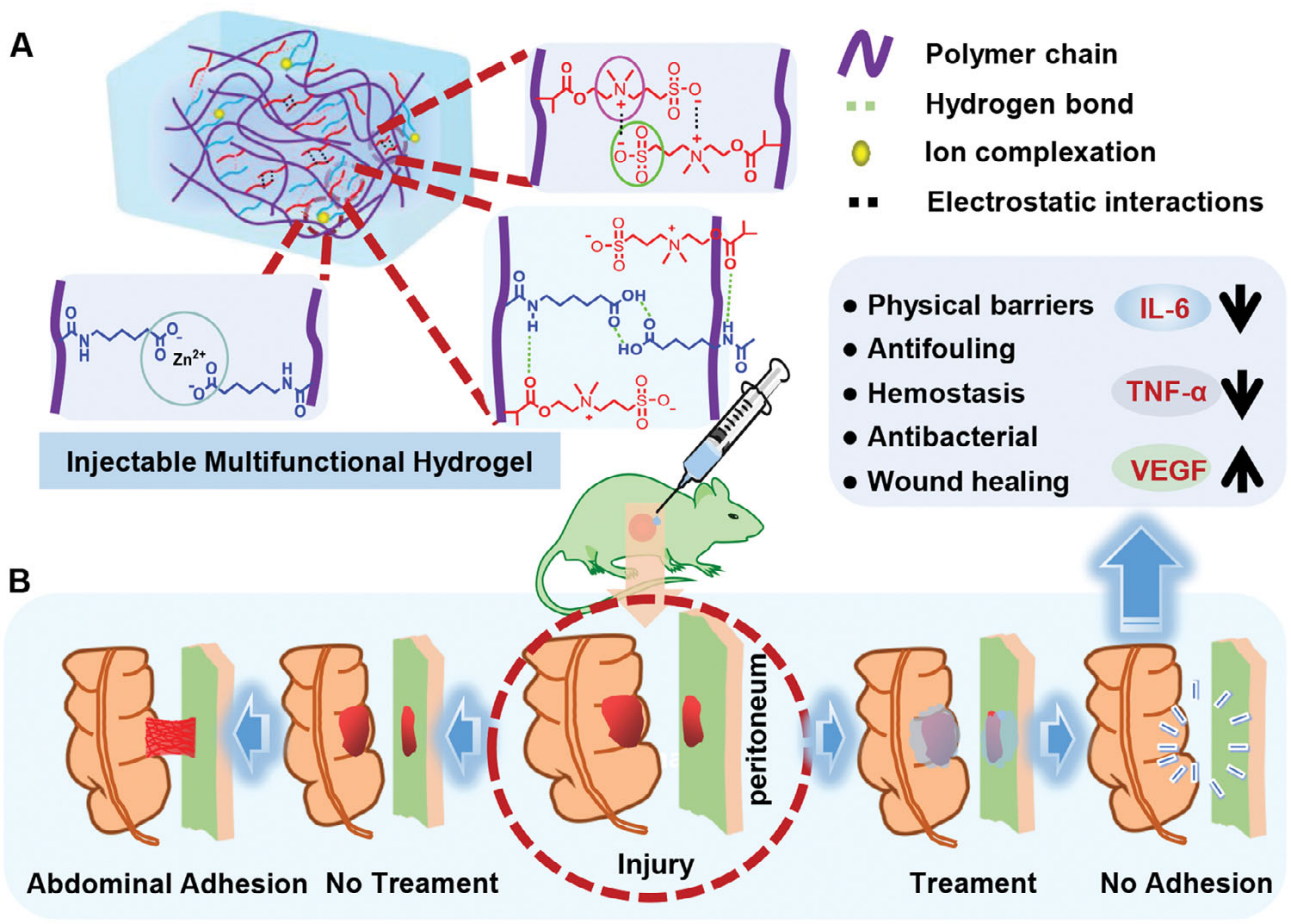
A) Schematic illustration of multiple intermolecular interactions within the PSA‐ZnO hydrogel. B) PSA‐ZnO hydrogel on rat cecum‐abdominal wall adhesion model.

Herein, we present a novel “one‐stone‐two‐birds” strategy that uniquely integrates these three functionalities into a single, injectable hydrogel platform (PSA‐ZnO) designed to overcome the limitations of existing barriers. Crucially, the components form an intertwined network stabilized by multiple weak interactions (e.g., hydrogen bonding, electrostatic forces, Zn^2^⁺ coordination; **Scheme** **1**A), enabling facile injection and resistance to abdominal stress. The hydrogel simultaneously delivers antibacterial, hemostatic, antifouling, and pro‐healing properties (Scheme 1B), rendering it highly effective against postoperative adhesions. In rat sidewall defect‐cecum abrasion models, PSA‐ZnO outperformed commercial hyaluronic acid (HA) gels by:(i) Acting as a physical barrier that reduces fibrin deposition and fibroblast adhesion,(ii) Adhering tightly to tissues to exert hemostasis and minimize bleeding‐induced fibrin accumulation, and(iii) Releasing Zn^2^⁺ controllably to enhance antibacterial activity, suppress inflammation, and accelerate wound healing. Collectively, this study presents an effective strategy for completely preventing postoperative abdominal adhesions and for inhibiting inflammation.

## 2 Results and Discussion

### 2.1 PSA‐ZnO Hydrogel Synthesis and Characterization

**Figure 1:**
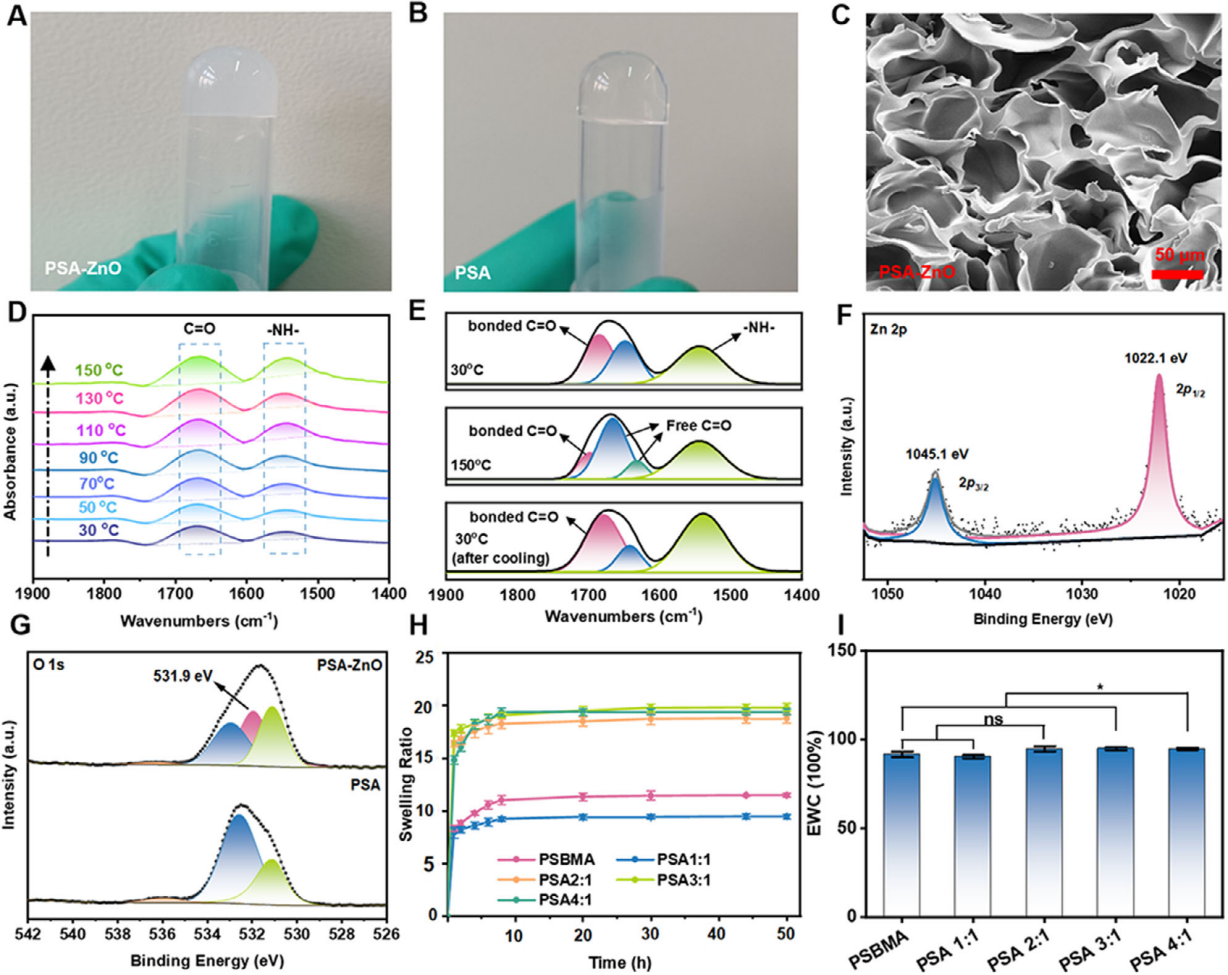
Formation and characterization of hydrogels. Photos of A) PSA‐ZnO and B) PSA hydrogels. C) SEM of PSA‐ZnO hydrogel. D) The heating process of variable‐temperature FT‐IR spectra of PSA hydrogel. E) Curve fitting of the carbonyl stretching region of PSA hydrogel powers at 30 °C, 150 °C, and cooled to 30 °C, respectively. The XPS spectrum of PSA and PSA‐ZnO hydrogel F) Zn 2p and G) O 1s. H) Swelling properties of PSA hydrogels with different ratios. I) Water content of PSA hydrogels with different ratios.

The PSA‐ZnO hydrogel was synthesized by dissolving SBMA, A6ACA, and ZnO nanoparticles (NPs) in deionized water, following polymerization (detailed preparation method in experimental section). The suspension was then converted into a gel at 50 °C (**Figure** **1**A). The hydrogel synthesized without ZnO NPs was designated as the parent PSA hydrogel (Figure 1B). The PSA‐ZnO hydrogel has excellent injection properties and is convenient for use in surgical procedures (Figure S1A, Supporting Information). The lyophilized and powdered hydrogel can be reconstituted in water (Figure S1B, Supporting Information). This is may be due to the intermolecular weak interactions between water and the good hydrophilicity of carboxyl groups, amide groups, and positive and negative charges in the hydrogel structure. This increases the potential of the hydrogel for various biomedical applications. The porosity and surface morphology of the PSA and PSA‐ZnO hydrogels were examined by scanning electron microscopy (SEM) and transmission electron microscopy (TEM), respectively. Both the PSA and PSA‐ZnO hydrogels exhibited a uniform and regular 3D porous structure with pores ranging from 10–50 µm (Figures 1C; S2A,B, Supporting Information). The pore size of the hydrogel increased with the SBMA content (Figure S3, Supporting Information). This may be because the highly charged SBMA improves the hydrophilicity of the hydrogel network structure and increases the ability of the hydrogel to bind water, thereby increasing the pore size. The unique porous structure may contribute to the reduction of the inflammatory response by absorbing the exudate from the wound.^[^ ^1^, ^5^ ^]^ The successful encapsulation of ZnO NPs in the hydrogel was also confirmed by both energy dispersive spectrometer (EDS) analysis and X‐ray diffraction (XRD) analysis. The ZnO NPs were well dispersed with a size of ≈50±10 nm (Figure S4, Supporting Information). Based on the elemental distribution, the elements C, N, O, S, and Zn were uniformly distributed in the PSA hydrogel (Figure S5A,B, Supporting Information). The XRD spectrum of the PSA‐ZnO hydrogel in the Figure S6 (Supporting Information) showed seven sharp diffraction peaks in (100), (002), (101), (102), (110), (103), and (112), indicating the crystallinity of the ZnO NPs in contrast to the amorphous of the parent PSA hydrogel.^[^ ^27^ ^]^

Furthermore, Fourier‐transform infrared spectroscopy (FTIR) and X‐ray photoelectron spectroscopy (XPS) were also performed for surface elemental composition and chemical structure characterization. The FTIR spectra confirmed that the parent PSA hydrogels were formed by copolymerization of SBMA and A6ACA, as the PSA hydrogels exhibited characteristic absorption peaks for ─C─N at 1548 cm^−1^ and for sulfonic acid groups at 1172 and 1040 cm^−1^, respectively (Figure S7A,B, Supporting Information). Also, the shift of the bands ≈1721 and 1668 cm^−1^, which are attributed to the carbonyl/carboxyl groups, respectively, is observed in the PSA. This is attributed to the formation of cross‐links due to the formation of H‐bond interactions between polymer chains (Figure S7C, Supporting Information). To verify this speculation, variable temperature FTIR absorption curves were used to determine the temperature response of intermolecular weak interactions in PSA hydrogel. As the temperature increases from 30 to 150 °C, the C═O stretching signal of the PSA hydrogel shifts from 1672 cm^−1^ to lower wave numbers (1664 cm^−1^), while the N─H bending signals (1548 cm^−1^) shift to wave numbers (1540 cm^−1^) (Figure 1D). The FT‐IR spectrum of the PSA hydrogels collected during cooling is almost identical to that of the PSA hydrogels collected during heating (Figure S8, Supporting Information). These shifts are caused by H‐bonding dissociation between N─H and C═O species.^[^ ^2^ ^]^ Curve‐fitting of the derived carbonyl stretch region of the PSA hydrogels was also carried out (Figure 1E). The distinct characteristic peak at 1685 cm^−1^ is attributed to the bonded C═O groups, while the characteristic peaks at 1648 and 1632 cm^−1^ are attributed to the free C═O groups. With increasing temperature, the former peak strength decreases while the latter increases. These results also indicate that H‐bonding occurs in the hydrogel. Furthermore, the XPS survey spectrum showed that obvious Zn peaks appeared after incorporating ZnO particles into the PSA‐ZnO hydrogel (Figure S9, Supporting Information). The binding energies of Zn 2p_3/2_ and 2p_1/2_ were located at 1022.1 and 1045.1 eV in the Zn 2p spectrum, which confirmed the existence of Zn^2+^ (Figure 1F). Furthermore, the characteristic peak of O─Zn─O appeared in the O 1s spectrum of PSA‐ZnO hydrogels (Figure 1G), indicating that Zn species easily complexed with the carboxylate groups of aminocaproic acid via electrostatic interactions.

The swelling performance of the PSA hydrogel improved with increasing SBMA content in Figure 1H, and the maximum swelling ratio was 19.84 when the ratio of SBMA to A6ACA was 3:1, which may be influenced by the intermolecular interaction between the zwitterion and other functional groups (such as carboxyl group, carbonyl group, etc.) in the PSA chain. As shown in Figure 1I, the water content of all these hydrogels is above 90.0%, with the highest water content of PSA 3:1 being 95.1%. The PSA‐ZnO hydrogel can effectively integrate intertwined intermolecular interactions, including hydrogen bonding, electrostatic interactions, and polymeric interactions, to synergistically enhance its remarkable combination of hydration ability.^[^ ^28^ ^]^ Appropriate in vivo retention time of hydrogels is essential for their application as antifouling biomaterials.^[^ ^14^, ^17^ ^]^ In vitro degradation studies (Figure S10, Supporting Information) revealed that after 20 days in PBS (pH 7.4), hydrogels exhibited sustained mass loss (8–22%), consistent with gradual erosion. The PSA hydrogel demonstrated enhanced stability versus PSBMA, attributable to hydrogen‐bond reinforcement from A6ACA monomers – a key aspect of our multi‐interaction network. Notably, PSA‐ZnO displayed the slowest degradation kinetics due to synergistic interactions: Zn^2^⁺ coordination bonds with zwitterionic moieties (‐COO−/‐SO^3;−^) formed hydrolysis‐resistant complexes, while electrostatic/hydrophobic interactions further stabilized the matrix. This orchestrated degradation profile ensures clinically adapted retention (7–14 days), fully covering the critical fibrotic phase of adhesion formation before programmed clearance.

### 2.2 Rheological Behaviors of Hydrogel

**Figure 2:**
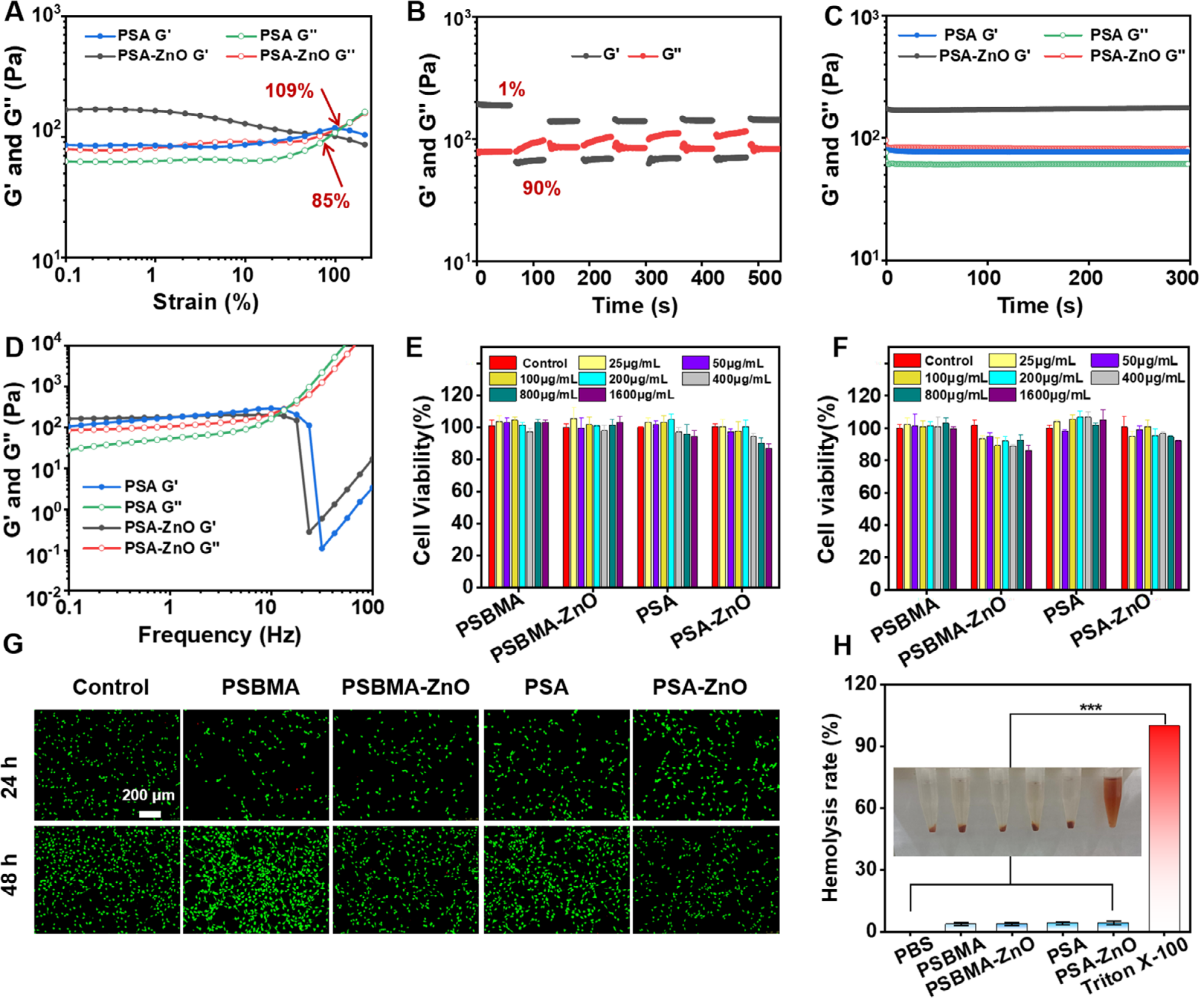
Rheological behavior and biocompatibility of hydrogel. A) Strain (from 0.1% to 200%) sweep at a frequency of 1 Hz. B) Step strain‐time sweep at 1% of strain for 60 s and 90% of strain for 60 s for five consecutive cycles. C) Time sweep at 1% of strain and 1 Hz of frequency for 300 s. D) Frequency sweep from 1 to 100 Hz at 1% of strain. Hydrogel extracts were co‐cultured with NIH‐3T3 cells for E) 24 h and F) 48 h for cell activity. G) Live/dead staining of NIH 3T3 cells after incubation with hydrogels for 24 and 48 h, respectively. H) Hemolysis test of hydrogels.

The rheological behavior of the parent PSA and PSA‐ZnO hydrogels was evaluated using a rheometer (MCR 302, Anton‐Paar, Austria). First, strain sweep tests were performed on the hydrogels, considering the application of these hydrogels in the rat abdomen and the damage to the hydrogels caused by abdominal movements (**Figure** **2**A). Both the parent PSA and PSA‐ZnO hydrogels exhibited stable mechanical properties in the early deformation phase, followed by a decrease in *G'* and an increase in *G“”*, thus showing crossover. The crossover point indicated that the viscoelastic behavior of the hydrogel changed from gel to solution, which was caused by the disruption of the internal structure of the hydrogel and fracture. In particular, a maximum *G'* (167.35 Pa) for the PSA‐ZnO hydrogel is higher than a maximum *G'* (85.48 Pa) for the PSA hydrogel. This may be due to the cross‐linking of Zn^2+^ with 6‐aminocaproic acid of the polymer chains through electrostatic interactions between Zn^2+^ and carboxylate groups, which allows extracellular proteins for better tissue adaptation, thus increasing the modulus of the PSA‐ZnO hydrogel.^[^ ^30^, ^31^, ^32^ ^]^ In addition, the PSA‐ZnO hydrogel had an earlier crossover point (85%) than the PSA hydrogel (109%). This may be due to the increase in hydrogel modulus by ZnO NPs while increasing the brittleness of the hydrogel. Moreover, since in vivo tissues experience ≈10% of the maximum strain, ^23^ a PSA‐ZnO hydrogel that could withstand 85% of the maximum strain was sufficient to maintain integrity in vivo. Next, a step strain‐time sweep of the PSA‐ZnO hydrogel was performed to investigate the self‐healing performance of the hydrogel, with a minimum strain of 1% and a maximum strain of 90%. As shown in Figure 2B and *G'* < *G“”* of the hydrogel at a high strain of 90% was the solution state; *G'* > *G“”* of the hydrogel at a low strain of 1% changed back to the gel state again. This indicated that the PSA‐ZnO hydrogel had self‐healing properties and was capable of retransforming back into a gel after syringe injection. The time sweep in Figure 2C showed that the hydrogel was stable. The frequency sweep results were shown in Figure 2D, where the hydrogel could maintain the gel state at low frequencies, and the internal structure of the hydrogel was destroyed at high frequencies.

### 2.3 Biocompatibility of PSA‐ZnO Hydrogel

A prerequisite for use as a biomaterial was the need for good biocompatibility of the material.^[^ ^33^ ^]^ In vitro hydrogel cytotoxicity was tested using mouse embryonic fibroblasts (NIH‐3T3) co‐cultured with a series of hydrogel extract concentration gradients (Figure 2E,F). The cell survival rate of all hydrogel groups was over 80%. This demonstrated the excellent cytocompatibility of the hydrogels. After live/dead cell staining, it could be seen that most cells were green with normal morphology, laterally confirming the hydrogel cytocompatibility (Figure 2G). In addition, the blood compatibility of the material must be considered when hydrogels are used as biomaterials in vivo. Hemocompatibility of hydrogels was tested with rat erythrocyte suspensions and hydrogel extracts. As shown in Figure 2H, the hemolysis rates of all hydrogel groups were less than 5%, indicating that the hydrogels had good hemocompatibility.^[^ ^34^ ^]^ Thus, the PSA‐znO hydrogel is biocompatible and safe for subsequent in vivo experiments.

### 2.4 In Vitro Antibacterial Activity and Zn2+ Release

**Figure 3:**
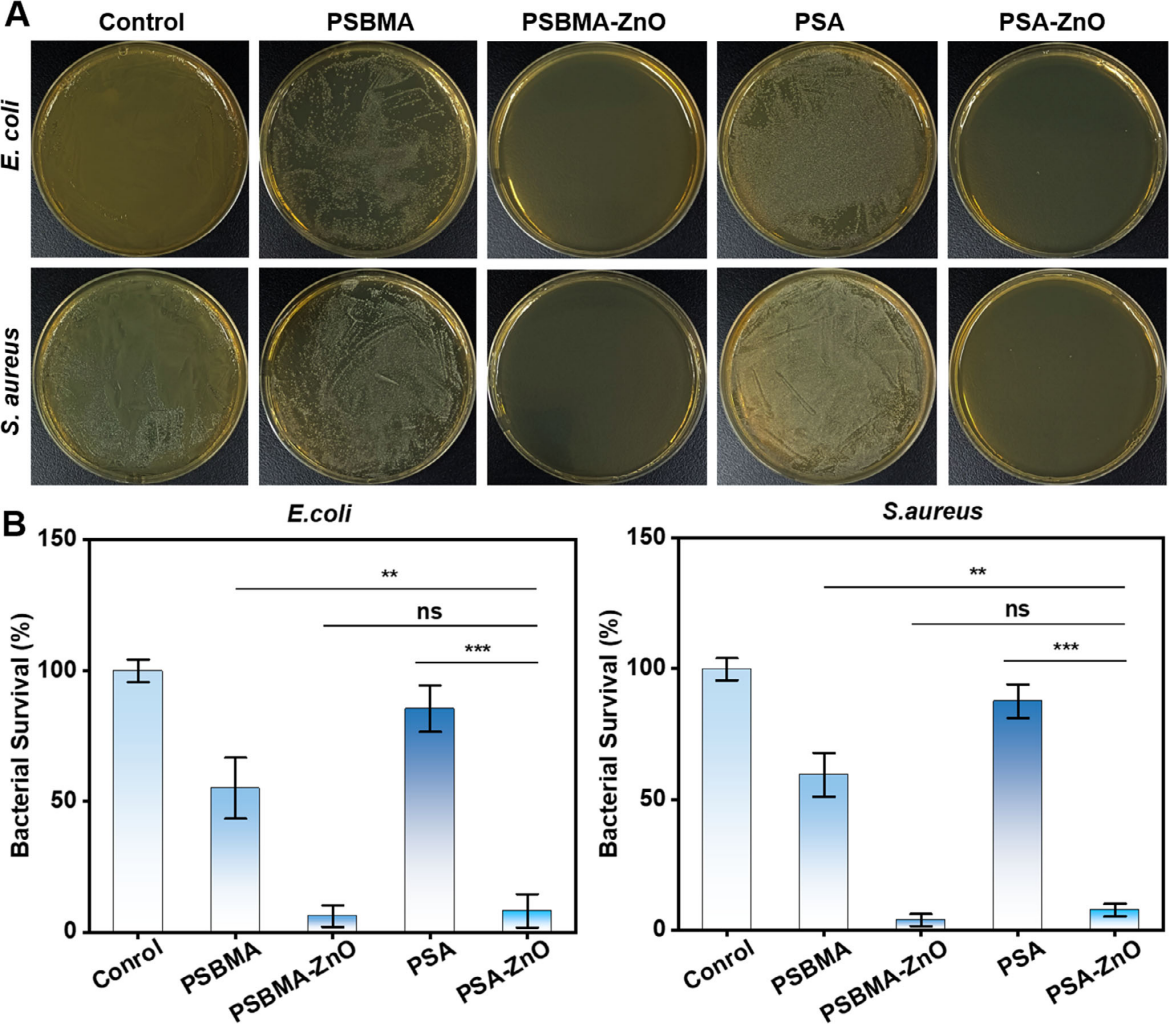
Antibacterial activity of hydrogels against *E. coli* and *S. aureus*. A) Optical images of hydrogels on agar plates. B) Quantitative analysis of bactericidal activity.

Hydrogels with antimicrobial properties are important in preventing peritoneal adhesions because bacterial infections of the peritoneal cavity produce both inflammation and stimulation of the review on epidermal growth factor receptor (EGFR) signaling in mesothelial cells to exacerbate peritoneal adhesions.^[^ ^35^ ^]^ The antibacterial properties of the hydrogels were verified using *Escherichia coli* (*E. coli*) and *Staphylococcus aureus* (*S. aureus*). It can be seen that there is a growth of bacteria all over the agar medium of the control group. For the parent hydrogels (PSBMA and PSA), the number of bacteria on the plates is slightly decreased. The colonies on the PSBMA‐ZnO and PSA‐ZnO hydrogel plates doped with ZnO NPs is almost negligible (**Figure** **3**A) and showed more than 95% antibacterial efficacy against both *E. coli* and *S. aureus* (Figure 3B). The release of Zn^2+^ ions from the ZnO NPs is one of the main factors for improving their antibacterial activity.

ZnO NPs have excellent antibacterial properties. Small amounts of Zn^2+^ could promote angiogenesis and accelerate wound healing. However, excessive doses of ZnO may be toxic to the body. Therefore, the amount of ZnO should be strictly controlled. The Zn^2+^ release profile of PSA is quantified using atomic absorption spectrophotometry and shows that ≈16% of the Zn^2+^ was released within 24 h and ≈53% within 150 h (Figure S11, Supporting Information). This demonstrates that the PSA‐ZnO hydrogel could sustain the release of ZnO to maintain the long‐term antimicrobial effect. This is sufficient to support the wound until healing.

### 2.5 In Vitro Antifouling Capability of Hydrogel

**Figure 4:**
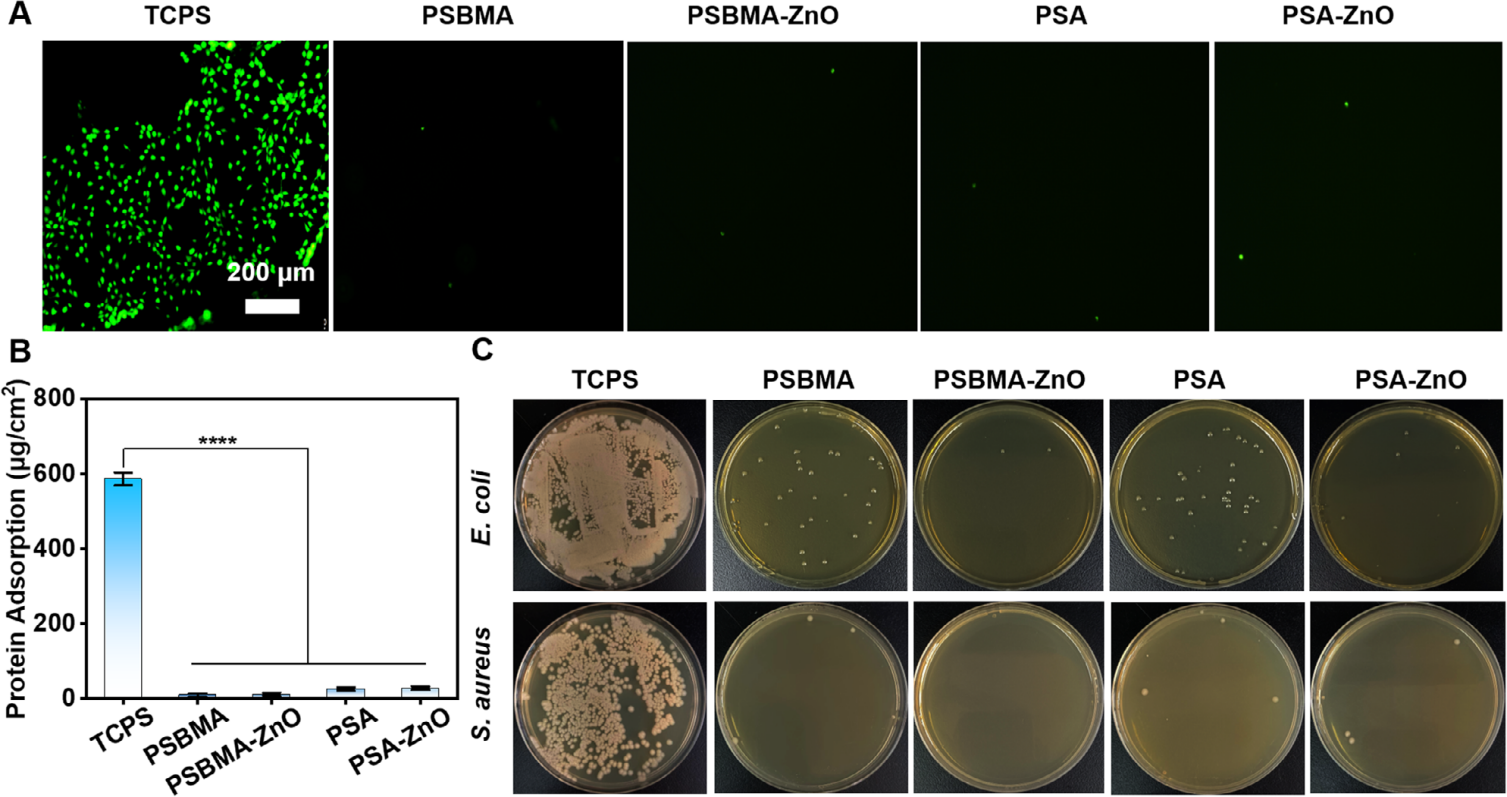
The antifouling properties of hydrogels against *E. coli* and *S. aureus*. A) Cell adsorption resistance (fluorescence imaging). Scale bar: 200 µm. B) Protein adsorption resistance (quantitative analysis). C) Bacterial colonization on surfaces (agar plate assay).

The antifouling properties of hydrogels at the biological level are usually expressed in terms of resistance to the adsorption of proteins, cells, and microorganisms.^[^ ^36^ ^]^ First, the foreign body reaction of hydrogels is a major concern when they are implanted as biomaterials in the body. On the other hand, many studies have considered the adsorption of fibrin and fibroblasts at the wound site after laparotomy as an essential factor that contributes to the postoperative tissue adhesions.^[^ ^37^, ^38^ ^]^ Thus, the antifouling properties of the hydrogels are tested using non‐specific proteins, cells, and bacteria in contact with the hydrogels. A comparison of cell staining results between standard tissue culture polystyrene (TCPS) and the hydrogel groups is shown in **Figure** **4**A. More cells were adsorbed on the surface of TCPS, while hydrogel groups showed almost no adsorption of cells. Furthermore, the protein adsorption study was performed using a standard protein adsorption protocol (Meilunbio, Dalian, China). Compared with that of TCPS (587 µg∙cm^−2^), the protein adsorption amount of hydrogel groups was less than 30 µg∙cm^−2^ (Figure 4B). The bacterial adhesion of the hydrogel groups was studied using *E. coli* and *S. aureus* as a model bacterium. As shown in Figure 4C, the number of colonies on the agar plates of the hydrogel groups was significantly reduced compared to the TCPS control group. The PSA‐ZnO hydrogel had the lowest number of colonies. These results indicate that the PSA‐ZnO hydrogels exhibit excellent antifouling properties. The anionic and cationic hydrophilicity of zwitterionic hydrogel polymers allows the formation of a surface hydration layer that prevents the adsorption of various pollutants.^[^ ^16^, ^17^, ^18^ ^]^ The antifouling of hydrogels is synergistic with the bactericidal ability to enhance the antibacterial adhesion properties of hydrogels. Therefore, this hydrogel possesses excellent potential for application toward abdominal adhesions.

### 2.6 In Vivo Hemostatic Capacity of PSA‐ZnO Hydrogel

Hemostats are also important in the prevention of abdominal adhesions. This is because bleeding and coagulation affect tissue adhesion. Postoperative bleeding at the site of peritoneal injury results in clotting and insoluble fibrin, which typically promotes fibroblast proliferation and the formation and development of adhesions. Adhesions will continue to form as the foreign body exacerbates the inflammatory response.^[^ ^31^ ^]^ A rat liver model is used to evaluate the hemostatic properties of the PSA‐ZnO hydrogel in vivo (Figure S12A, Supporting Information).^[^ ^39^ ^]^ The bleeding liver was punctured with a 20 G needle, the bleeding volume was recorded within 2 min, and the time when the blood no longer exuded was recorded. As shown in Figure S12B (Supporting Information), a large amount of blood is left on the filter paper in the control group, and the blood on the filter paper is lighter in the PSA‐ZnO hydrogel group. The quantitative results show that the hemostasis time of rats in the control group was ≈109 s and the bleeding volume was ≈195 mg, while PSA‐ZnO hydrogel injection treatment rats had a hemostasis time of ≈55 s and a hemorrhage volume of ≈54 mg (Figure S12C, Supporting Information). These results indicate that the PSA‐ZnO hydrogel exhibits a better hemostasis in vivo, which attributing to the porous structure of the hydrogel as observed by SEM (Figure 1C) and the intrinsic hemostasis activity of 6‐aminohexanoic acid.

### 2.7 In Vivo Wound Healing of PSA‐ZnO Hydrogel

**Figure 5:**
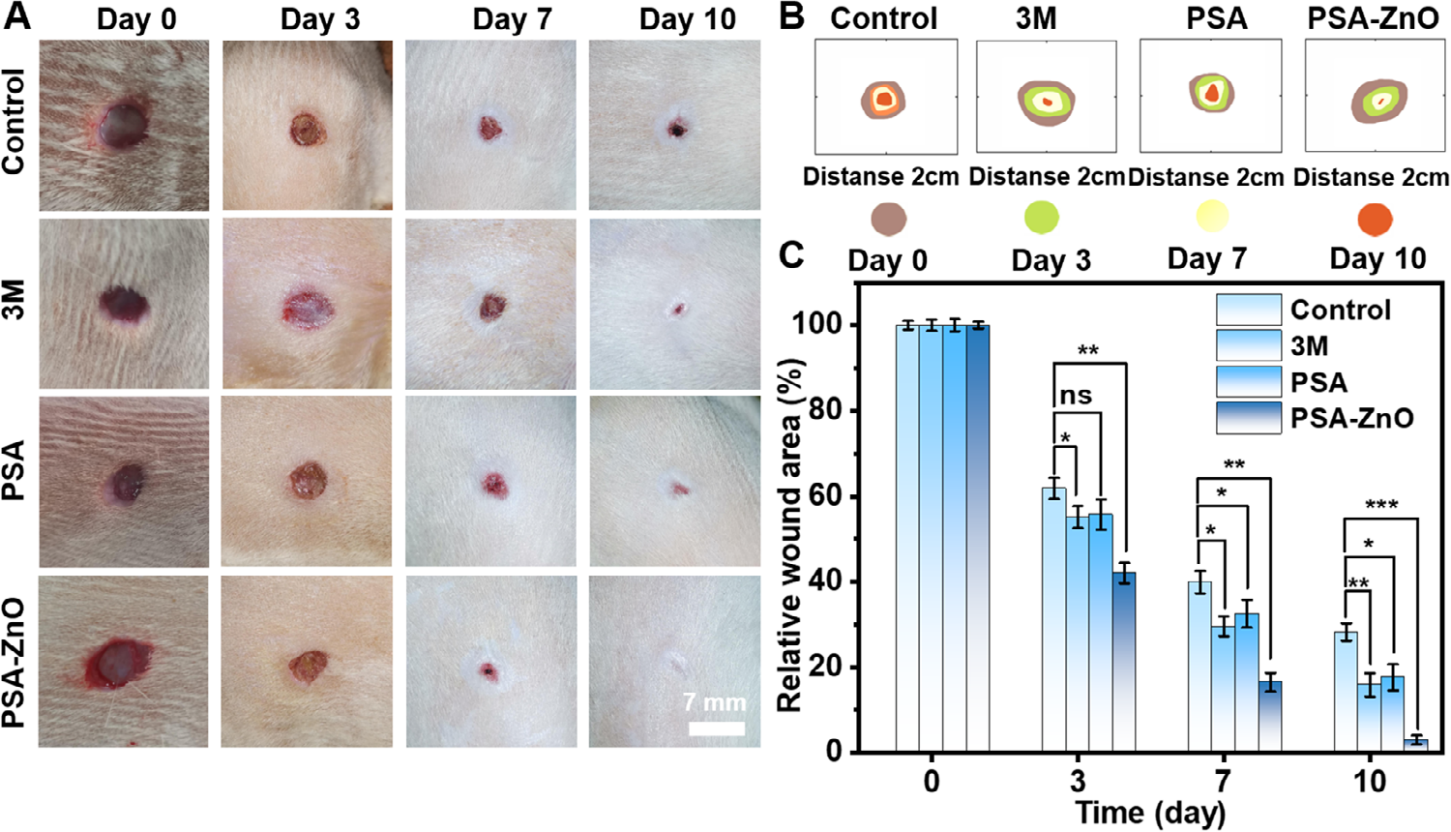
In vivo assessment of hydrogel wound healing in a rat full‐thickness skin wound model. A) Representative photographs of wound healing in the control, commercial 3 m dressing, PSA hydrogel, and PSA‐ZnO hydrogel groups at days 0, 3, 7, and 10. Scale bar: 7 mm. B) Changes in wound healing in each group. C) Relative wound area in each group at different times.

A rat full‐thickness skin wound model is used to evaluate the effect of hydrogels in promoting wound healing.^[^ ^40^ ^]^ The wound area of the PSA‐ZnO hydrogel group was smaller than that of the other groups on day 3 (**Figure** **5**A,B). It can be found that the wounds of both groups of rats, PSBMA and PSA‐ZnO hydrogel with zwitterionic ionic composition, were moist, which proved that the hydrogel could provide a good moist healing environment at the wound site. On day 7, the wound area in the PSA group was reduced to 16% of the initial area, much smaller than the wounds in the other groups. By day 10, the wounds in the PSA‐ZnO hydrogel group were almost completely healed, and the wound area was less than 5% of the initial wound area. The PSA‐ZnO hydrogel group showed the fastest healing rate throughout the healing process. This compares to 28% of the initial area in the control group, 16% of the initial area in the 3 m dressing group, and 18% of the initial area in the PSBMA hydrogel group (Figure 5C). However, even though the wounds in the other three groups are almost healed, the photographs show that there are still very obvious crusts in the control and 3 m dressing groups. This indicates that the wounds had not reached the state of complete healing and would eventually leave scarring. In contrast, the PSBMA hydrogel group shows no crusts in the wounds and the tissues are red in color, although the wound area had not shrunk to healing. For the PSA‐ZnO hydrogel group, wound healing is accelerated, and no scar is left after healing. This is due to the excellent antibacterial, antifouling, and hydrophilic properties of the hydrogel.

**Figure 6:**
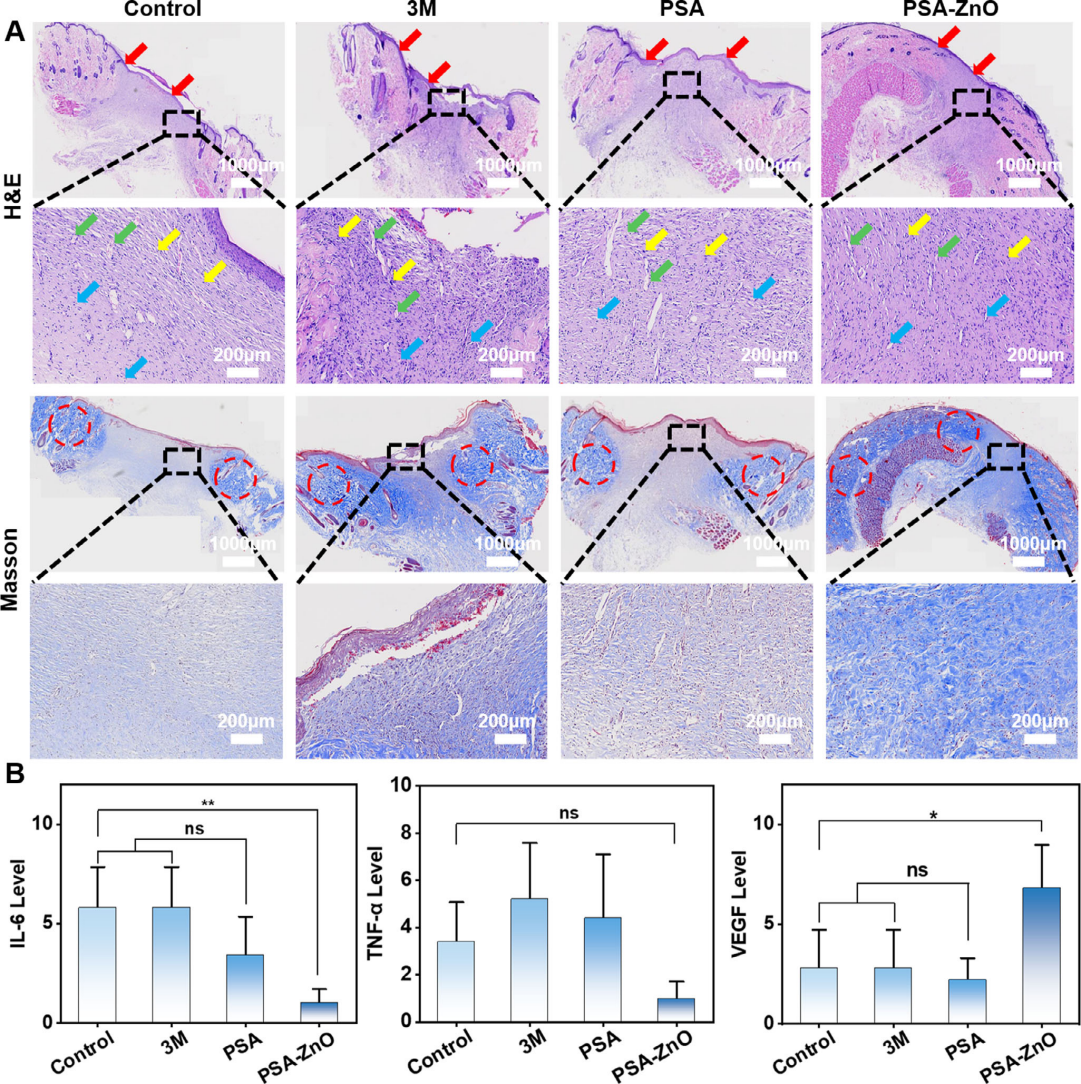
Wound histological evaluation of hydrogels. A)Histopathological analysis of wound tissues on day 10 by H&E staining and Masson trichrome staining. Red, green, blue, yellow arrows, and red circles are used to indicate the epithelial layer, granulation tissue, blood vessels, inflammatory cells, and collagen. Scale bar: 1000 µm. B)The image at the bottom is a partial enlargement of the image above. IL‐6, TNF‐α, and VEGF levels were assessed by immunohistochemical staining on day 10.

To further evaluate the healing of the incisions, hematoxylin and eosin (H&E) staining of the wound tissue is also performed on day 10 as shown in **Figure** **6**A. The PSA‐ZnO hydrogel group showed better epithelial remodeling, granulation tissue regeneration, and angiogenesis than the other groups. Additionally, collagen deposition in skin tissue was observed by masson's trichrome (MT) trichrome staining. Collagen was stained blue, while keratin and muscle fibers were stained red. Compared to the other groups, the PSA‐ZnO hydrogel group had higher collagen composition and more uniform deposition. Finally, immunohistochemical staining is used to evaluate the levels of pro‐inflammatory cytokines, interleukin 6 (IL‐6), tumor necrosis factor‐α (TNF‐α), and the growth factor, vascular endothelial growth factor (VEGF).^[^ ^41^, ^42^, ^43^ ^]^ IL‐6 and TNF‐α levels were significantly decreased and VEGF levels were massively increased in the PSA‐ZnO hydrogel group compared with the other three groups (Figures 6B; S13, Supporting Information). The results show that PSA‐ZnO hydrogel could inhibit inflammation, promote new blood vessel formation, and significantly accelerate the process of tissue regeneration and wound healing.

### 2.8 In Vivo Prevention of Postoperative Abdominal Adhesions of PSA‐ZnO Hydrogel

**Figure 7:**
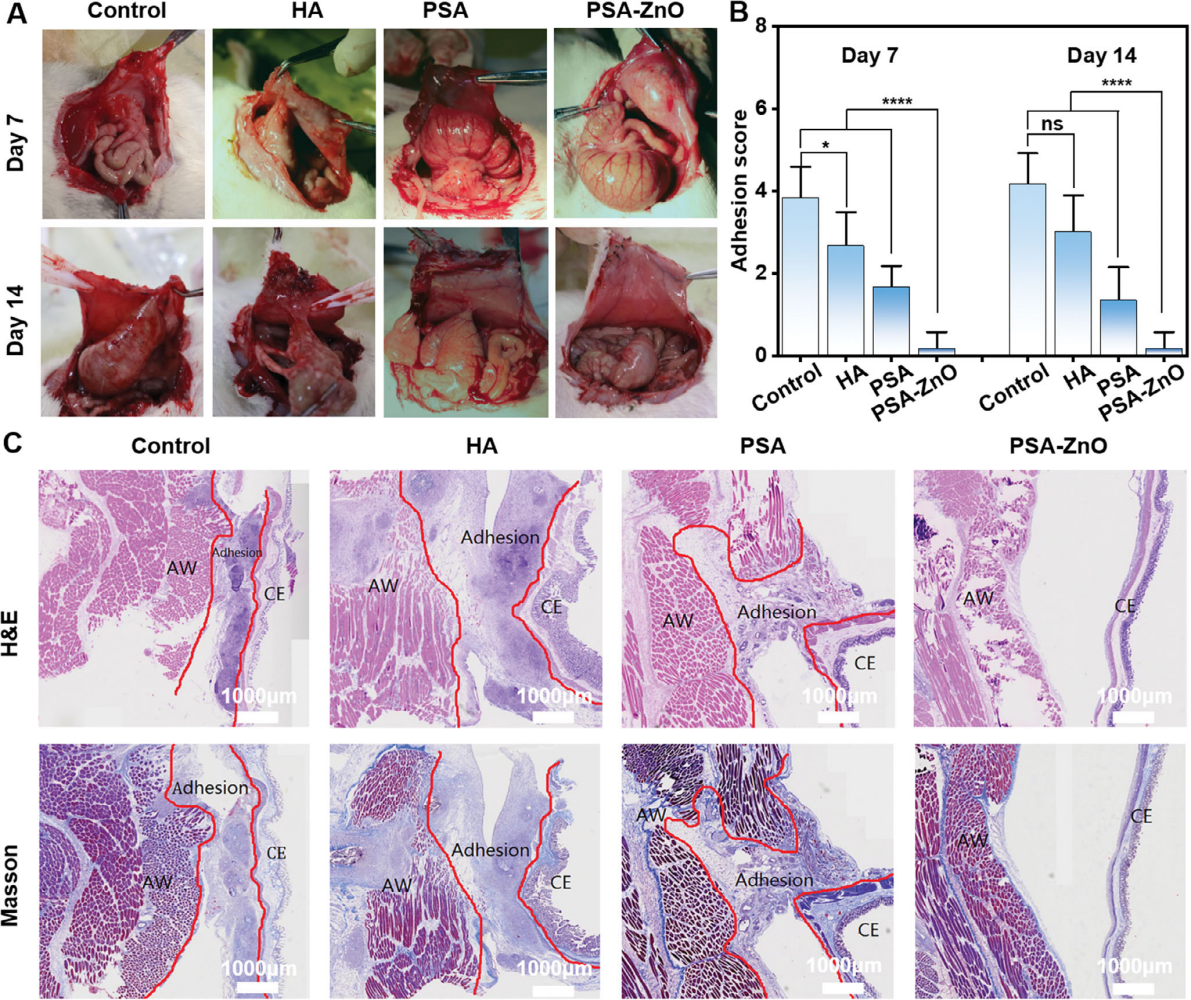
Postoperative abdominal adhesions effect of hydrogels. A) Representative pictures of abdominal adhesions in each group at day 7 and day 14 after treatment. B) Scores of abdominal adhesions in each group at day 7 and day 14 after treatment. C) Histopathological analysis of each group of specimens by H&E staining and MT staining on postoperative day 14. AW: abdominal wall. CE: cecum. Scale bar: 1000 µm.

The hydrogels were tested for postoperative antifouling using the rat sidewall defect cercal abrasion model.^[^ ^23^ ^]^ The rats with the cecum and peritoneal injury were randomly divided into four groups: blank control group, commercial HA gel group, PSA hydrogel group, and PSA‐ZnO hydrogel group, where the commercial HA gel group was the positive control group. The abdomens of the rats were opened on postoperative days 7 and 14. Adhesions in the abdominal cavity of the rats were observed and evaluated for scoring. As shown in **Figure** **7**A, the blank control group showed severe adhesions and more areas of adhesion on both days 7 and 14, consistent with previously reported results.^[^ ^44^ ^]^ On day 7 and day 14, the HA gel group still showed more adhesions, and the PSA hydrogel group showed better adhesions than the HA gel group, with fewer adhesions. This may be due to the easy degradation of the HA gel and its failure to adequately protect the damaged parts of the abdominal cavity prior to wound healing, which is also a drawback of such natural polymeric antifouling materials.^[^ ^45^, ^46^ ^]^ For the PSA‐ZnO gel group, no adhesions were observed on either day 7 or day 14. As shown in Figure 7B, the score for the PSA‐ZnO hydrogel group is significantly lower than that for the other three groups. In brief, these results indicate that the PSA‐ZnO hydrogel is significantly effective in the prevention of abdominal adhesion, and the superior antimicrobial, hemostatic, and pro‐wound healing properties have a synergistic effect in the prevention of abdominal adhesion and improvement of wound healing after surgery.

Furthermore, the cecum and corresponding abdominal wall tissues were collected from rats after 14 days and histologically analyzed using H&E staining and masson's trichrome staining. H&E staining revealed areas of adhesions/connective tissue between the muscular layer of the intestinal and abdominal walls with dense collagen bands and easily identifiable inflammatory cells in the blank control, HA commercial, and PSA groups (Figure 7C). An abundant collagen fibril deposition was observed by Masson staining in the three groups mentioned above. In the PSA‐ZnO group, there were no adhesions between the abdominal wall and the intestinal wall. The damaged part of the peritoneum was covered by a new layer of peritoneal mesothelial cells without collagen deposition. Furthermore, H&E staining results further confirmed that implantation of PSA‐ZnO hydrogel did not induce any significant pathologic changes in major organs, including heart, liver, lungs, and kidneys in rats after 30 days (Figure S14, Supporting Information). These results show that PSA‐ZnO hydrogel could better inhibit peritoneal adhesions and promote peritoneal repair in rats. The possible antifouling mechanism of PSA‐ZnO hydrogel is, when the injectable PSA‐ZnO hydrogel is injected to cover the abdominal wound in rats as a physical barrier to stop wound bleeding, reduce wound exudate based on forming a hydrated layer on the surface of the hydrogel to prevent various contaminants from adsorbing. Subsequently, the slow release of ZnO nanoparticles and Zn^2+^ enhanced the antibacterial properties of the hydrogel, reduced inflammation, and promoted wound healing, while the hydrogel could modulate the reduction and deposition of fibrin, prevent foreign body formation from thrombus, and adsorb and proliferate fibrin and fibroblasts in vivo. This synergistically prevents postoperative abdominal adhesions.

## 3 Conclusion

In summary, we developed an injectable zwitterionic hydrogel (PSA‐ZnO) via facile one‐pot synthesis, integrating synergistic antifouling, haemostatic, and antimicrobial functionalities. PSA‐ZnO demonstrated exceptional clinical efficacy in a rat sidewall defect‐cecum abrasion model, outperforming commercially available hyaluronic acid (HA) with a significant reduction in adhesion formation and complete prevention of pathological scar formation thanks to its remarkable antifouling effects. This multifunctional performance, combining broad‐spectrum antibacterial action, balanced haemostasis, and adhesion prevention, stems from its unique intermolecular architecture. FTIR and XPS analyses confirmed the presence of weak hydrogen bonding and electrostatic interactions between zwitterionic moieties and ZnO nanoparticles, which are easily injectable and resistant to abdominal creep stress. This effectively prevents foreign body formation from blood clots and insoluble fibrin at the wound site. This work provides a promising and effective biomaterial for preventing postoperative adhesions and reducing inflammation caused by bacterial infection and a foreign body reaction in vivo, with tremendous potential for practical applications.

## 4 Experimental Section

### Experimental Materials

N‐(3‐Sulfopropyl)‐N‐Methacryloxyethyl‐N, N‐Dimethylammonium Betaine (SBMA) was purchased from Shanghai Titan Scientific Co., Ltd (Shanghai, China). Ammonium persulfate (APS) was purchased from Sinopharm Chemical Reagent Co., Ltd (Shanghai, China). Acryloyl chloride, 6‐aminocaproic acid, N, N’‐methylene bisacrylamide (BIS), tetramethylethylenediamine (TEMED), and zinc oxide nanoparticles were purchased from Shanghai Aladdin Biochemical Technology Co., Ltd (Shanghai, China). The Sprague–Dawley (Male, 180–220 g) rats used in the experiments were provided by Wu Laboratory Animals (Fujian, China). All reagents were analytical grade and could be used directly. All animal experiments were approved by the Animal Ethics Committee of the Fujian Provincial Hospital (FPH.PZ.20230506 [0001]) and carried out in accordance with the Guidelines of the Animal Care and Use Committee of the establishment.

### Synthesis of A6ACA

Acryloyl chloride grafted 6‐aminohexanoic acid (A6ACA) was prepared according to a previous literature report ^28^. Briefly, 3.28 g of 6‐aminohexanoic acid, 1 g of NaOH, and 10 mL of ether were first weighed into a beaker with 50 mL of deionized water and stirred well by a magnetic stirrer, after which 2 mL of acryloyl chloride and 10 mL of ether were mixed well. The above mixture was added drop by drop in an ice bath and the reaction was carried out at room temperature for 4 h. After the reaction, the pH of the reaction solution was adjusted to ≈10 with 2 m NaOH and extracted with ethyl acetate, followed by adjusting the pH of the aqueous solution after the extraction with 6 m HCl to ≈2 and continuing the extraction with ethyl acetate. The product of acryloyl chloride grafted 6‐aminohexanoic acid was obtained by filtration after absorbing water with anhydrous sodium sulfate and rotary evaporation, and finally precipitated in petroleum ether.

### Preparation of PSA‐ZnO Hydrogel

PSA‐ZnO hydrogels were prepared as follows: 1120 mg of SBMA, 185 mg of A6ACA, 30 mg of N, N'‐methylene bisacrylamide (BIS), 10 mg of ZnO NPs were dissolved in 10 mL of deionized water, and the suspension was mixed well by ultrasonication and magnetic stirring, and then 10 mg of the initiator Ammonium persulfate (APS) and 150 µL tetramethylethylenediamine (TEMED) were added to the above suspension, and the PSA‐ZnO hydrogel was prepared by thermal initiation of free radical polymerization at 50 °C.

### In Vivo Hemostatic Properties of Hydrogel

The in vivo hemostatic properties of the hydrogel were assessed using a rat liver hemorrhage model. Briefly, rats were first anesthetized by injection of pentobarbital sodium. The abdomen was opened to expose the liver, and pre‐weighed filter paper was placed under the liver. The liver is punctured with a 20‐G needle for bleeding. The hydrogel was injected into the bleeding site, and the amount of bleeding was recorded within 2 min, and the time to hemostasis was recorded when the bleeding stopped. Wounds without any treatment were used as controls. The experiment was repeated five times for each group.

### In Vivo Wound Healing Assay of Hydrogel

Wound healing ability of hydrogel was measured using the rat skin incision model. The rats were fasted from water and food the day before the experiment, weighed the next day, and anesthetized by intraperitoneal injection of pentobarbital sodium. The hair on the back of the rats was shaved, disinfected with alcohol, and a 7‐mm circular skin incision was created on the back of the rats using a 7 mm skin punch. Twenty rats were randomly divided into four groups of five rats each, and the wounds were treated with saline, 3 m wound dressing, PSBMA hydrogel, and PSA‐ZnO hydrogel, respectively. After treatment, the rats were kept in a single cage and provided with sufficient water and feed. Wound healing was observed daily and recorded with a digital camera, while the 3 m dressing and hydrogel were changed. Wound photos at 0, 3, 7, and 10 days were selected, and the size of the wound area was calculated using the software Image. The relative wound area was determined by the following equation: Relative wound area (%) = A_n_ / A_0_ × 100%, where A_n_ represented the area of the wound on day n and A_0_ represented the area of the wound on day 0. In addition, the rats were euthanized after observation of the wounds on the tenth day. The tissues were removed intact from the wounds on the back of the rats and fixed in a solution of 4% paraformaldehyde solution. These collected tissues were embedded in paraffin wax, made into sections, and subjected to H&E staining, Masson trichrome staining, and immunohistochemical staining.

### In Vivo Postoperative Antifouling Assay of Hydrogel

The hydrogel was tested for postoperative antifouling in the rat sidewall defect‐cecum abrasion model. Rats were fasted with water and food the day before the experiment, weighed the next day, and anesthetized by intraperitoneal injection of pentobarbital sodium. The abdomen of the rat was shaved and disinfected with alcohol, and a 4–5 cm incision was made along the midline of the abdominal wall using surgical scissors. The cecum was separated, and the surface of the cecum was gently rubbed with sterile gauze until blood appeared. Then, a 1 cm × 2 cm‐sized peritoneal defect was made in the corresponding abdominal wall with a scalpel. The 48 rats with cecum and peritoneal damage were randomly divided into four groups of 12 rats each and injected with 1 mL of saline, commercial HA gel, PSA hydrogel, and PSA‐ZnO hydrogel to cover the cecum and peritoneal defects, and named as the control group, HA group, PSA hydrogel group, and PSA‐ZnO hydrogel group, respectively. After that, the rats' feeding, activity, and defecation were observed daily and recorded. Six rats in each group were euthanized after 7 and 14 days of treatment, respectively, and then the abdomen was carefully opened to observe the abdominal adhesions. The experimental results were recorded using a digital camera with photographs, and the adhesions of the cecum and abdominal wall in the abdominal cavity of the rats were scored artificially using a standard scoring system. After euthanizing the rats and examining the abdominal adhesions, the tissues of the cecum and the abdominal wall of the rats at the corresponding locations were collected and fixed in 4% paraformaldehyde solution. The collected tissues were used for H&E staining and Masson trichrome staining to analyze the pathological changes in the cecum and abdominal wall after surgery. In addition, the major organs (heart, liver, lung, and kidney) of rats in the PSA ZnO hydrogel group were stained with H&E on the 30 day after abdominal surgery for histological evaluation.

### Statistical Analysis

All data were expressed as mean ± standard deviation (SD). A one‐way analysis of variance (ANOVA) test in GraphPad Prism version 8.3 software was used to analyze the statistical significance between multiple groups. Statistical significance was set at *p* < 0.05. Data were plotted using Origin 2019 software and GraphPad Prism 8.3 software.

### Ethics Approval and Consent to Participate

All animal experiments were approved by the Animal Ethics Committee of the Fujian Provincial Hospital (FPH.PZ.20230506 [0001]) and carried out in accordance with the Guidelines of the Animal Care and Use Committee of the establishment.

## Conflict of Interest

The authors declare no competing financial interest.

## Author Contributions

N.W. wrote the original draft and was responsible for methodology, investigation, formal analysis, data curation, conceptualization, funding acquisition, and project administration. Y.J. contributed to methodology, investigation, formal analysis, and data curation. Y.S. contributed to methodology, investigation, formal analysis, and data curation. J.Y. contributed to methodology, investigation, formal analysis, and data curation. J.L. reviewed and edited the writing and was responsible for supervision, resources, project administration, and funding acquisition. Y.W. contributed to methodology and project administration. X.Y. contributed to methodology and project administration. S.L. contributed to methodology and project administration. T.Z. reviewed and edited the writing and was responsible for supervision and resources. X.H. contributed to methodology and project administration.

## Supporting information



Supporting Information

## Data Availability

The data that support the findings of this study are available from the corresponding author upon reasonable request.
