# Supplementary material for: An Injectable Zwitterionic Hydrogels with Multiple Intermolecular Interactions for Effective Prevention of Abdominal Adhesions
Source: Adv Sci (Weinh). 2025 Aug 19;12(42):e11757. doi: 10.1002/advs.202511757 (PMC12622508; doi:10.1002/advs.202511757)
Supplement: Supplementary file 1 — Supporting Information [file ADVS-12-e11757-s001.docx]

Supporting Information

An Injectable Zwitterionic Hydrogels with Multiple Intermolecular Interactions for Effective Prevention of Abdominal Adhesions

Na Wen*, Yating Jiang, Yunhao Song, Jiachao Yang, Jinlin Long, Ying Wang, Xunbin Yu, Shiyun Lu, Tianhua Zhou*, Xueping Huang*

N. Wen, Y. Jiang, Y. Song, J. Yang,

College of Materials Science and Engineering, Fuzhou University, Fuzhou, 350116, China

E-mail: wennaa@fzu.edu.cn

Prof. T. Zhou

State Key Laboratory of Structural Chemistry, Fujian Institute of Research on the Structure of Matter Chinese Academy of Sciences, Fujian 350002, P. R. China

E-mail: thzhou@fjirsm.ac.cn

Prof. N. Wen, Prof. J. Long, Prof. Y. Wang, Prof. T. Zhou

State Key Lab of Photocatalysis on Energy and Environment, Fuzhou University, Fuzhou, 350108, China

X. Yu, S. Lu, X. Huang

Fuzhou University Affiliated Provincial Hospital, Fujian Province, Fuzhou, 350001, China

E-mail: hxuep@mail2.sysu.edu.cn

X. Yu, S. Lu, X. Huang

Shengli Clinical Medical College, Fujian Medical University, Fujian Province, Fuzhou, 350001, China

X. Yu,

Department of Pathology, Fujian Provincial Hospital, Fujian Province, Fuzhou, 350001, China

S. Lu, X. Huang

Department of Gastroenterology, Fujian Provincial Hospital, Fujian Province, Fuzhou, 350001, China

1. **Experimental Section**

**1.1 Characterizations of Hydrogel**

Fourier transform infrared spectroscopy (Nicolet 5700, Thermo Elemental, USA) was used to study the functional group changes of PSA-ZnO hydrogels and their constituents A6ACA and SBMA in the wavelength range of 400 cm^-1^-4000 cm^-1^. Variable temperature FTIR was employed to confirm the formation of hydrogen bonds in PSA hydrogel by recording the infrared absorption curves at 30, 60, 90 and 150 ℃. X-ray photoelectron spectroscopy (XPS) measurements for surface elemental characterization by ESCALAB 250 (Thermo Scientific). The crystalline condition of PSA-ZnO hydrogel powder was investigated by X-ray diffractometer (ULTIMA III, Rigaku Corporation, Japan). The microstructure of hydrogels was observed by scanning electron microscopy (SEM, Nova NanoSEM 230, FEI CZECH REPUBLIC S.R.O., China), transmission electron microscopy (TEM, Talos F200i, FEI). T, and EDS analysis was performed.

**1.2 Swelling Behaviors and Water Content of Hydrogel**

Each hydrogel sample was freeze-dried and weighed, put into saline at 37 °C for swelling, and the hydrogel was removed at a fixed time point, and the water on the surface of the hydrogel was blotted with filter paper and weighed for recording. The hydrogel swelling ratio (SR) was determined by the following equation: SR = (W_t_ - W_0_)/W_0_, where W_0_ and W_t_ represented the mass of the hydrogel after freeze-drying and after different times of swelling, respectively. The experiment was repeated three times.

Equilibrium water content (EWC) refer to the weight of water contained in the system when the hydrogel reached swelling equilibrium. The hydrogel samples were weighed after freeze-drying and put into saline at 37 °C for swelling. When the hydrogel was completely swollen, the hydrogel was removed and the water on the surface of the hydrogel was blotted out with filter paper and weighed. The final mass needed to be measured several times, and when it no longer changed, it meant the hydrogel reached the equilibrium of swelling. The equilibrium water content of the hydrogel was determined by the following equation: EWC (%) = (W_s_ - W_0_)/W_s_ × 100 %, where W_0_ and W_s_ represented the mass of the hydrogel after freeze-drying and after reaching swelling equilibrium, respectively. The experiment was repeated three times.

**1.3 *In vitro* Degradation of Hydrogel**

To test the degradation properties of the hydrogels, the freeze-dried hydrogel samples were placed in 30 mL of pH=7.4 PBS buffer solution at 37 °C. The samples were removed at each fixed time interval (1, 3, 6, 10, 15, and 21 days), and the hydrogels were rinsed with deionized water. The hydrogel samples were lyophilized and weighed and recorded. The rate of hydrogel degradation was determined by the following equation: Degradation rate (%) = W_t_ /W_0_ × 100 %, where W_0_ and W_t_ represented the mass of the initial post-freeze-dried hydrogel sample and the remaining post-freeze-dried hydrogel sample after degradation at different time intervals, respectively. The experiment was repeated three times.

**1.4 Rheological Behaviors of Hydrogel**

Rheological testing of hydrogels was performed using MCR 302 rheometer (Anton-Paar, Austria) equipped with 25mm parallel plates at 25 °C. A strain sweep (1%-200%) was first performed to determine the linear viscoelastic regime of the hydrogel. Then step-strain time sweep, time sweep, and frequency sweep were performed. In the step strain-time sweep test, the time test was performed at a constant frequency of 1 Hz, alternating between 1% minimum strain (1 min) and 90% maximum strain (1 min) for five cycles. The time sweep test was performed at 1 Hz and 1% strain for 300 s. The frequency sweep test was performed at 1% strain over a frequency range of 0.1 to 100 Hz.

**1.5 Biocompatibility of Hydrogel.**

NIH-3T3 cells were cultured in DMEM complete medium (90% DMEM, 10% FBS, 1% penicillin-streptomycin), inoculated in T25 cell culture flasks, and placed in a 37 °C, 5% CO_2_ cell culture incubator. The cell growth was observed daily by inverted microscope (DMIL LED, Leica), and when the cells grew to more than 80%, they were diluted to the desired concentration after cell counting and inoculated into 96-well plates at a cell density of 1×10^4^/well, and after 24 h of culture, the medium in the well plates was discarded and replaced with an equal amount of gel extract as the experimental group, and the blank group was replaced with fresh cell culture medium. After incubating for 24 h and 48 h, 10 μL of CCK-8 solution was added and incubated at 37 °C for 4 h. The absorbance at wavelength 450 nm was measured using a microplate reader (SpectraMax ID5, Molecular Devices). Six parallel wells were set up for each group. Cell viability was determined by the following equation: Cell viability (%) = (OD_sample_ - OD_blank_)/(OD_control_ - OD_blank_) × 100%, where OD_sample_ represented the absorbance of sample wells with gel dip added, OD_control_ represents the absorbance of control wells without gel dip added, and OD_blank_ represents the absorbance of background wells with only culture medium, CCK-8 solution.

To observe the cytotoxicity of hydrogels more visually, live/dead cells were stained using a live/dead cell staining kit. Briefly, 3mL of cell suspension at a concentration of 1×10^5^ mL^-1^ was added to a 35 mm culture dish and incubated in the cell culture dish for 24 h, then medium was discarded and replaced with gel infusion solution. After continuing to incubate for 24 h and 48 h, removed the medium and then washed gently with PBS buffer solution 2-3 times, added the configured cell staining solution and incubated for 20 min at 37 °C. Observed the cell images under an inverted fluorescence microscope (DMI8, Leica) and taken pictures for recording.

The hydrogel extract was obtained by adding 1 mL of hydrogel sample to 10 mL of saline and incubating at 37 ℃ for 48 h. Rat blood was taken and dissolved in saline, and erythrocytes were obtained by centrifugation (2000 rpm/min, 5 min). The obtained erythrocytes were diluted with saline to 5 % for use. The gel extract and 5 % erythrocyte suspension were mixed in equal volumes and incubated at 37 ℃ for 3 h, followed by centrifugation (2000 rpm/min, 5 min), and the experimental results were observed and photographed, and recorded. Another 200 μL of supernatant was collected, the absorbance at wavelength 545 nm was measured using a microplate reader, and the hemolysis rate of each group was calculated by absorbance. Physiological saline was used as the negative control group, and Triton X-100 was used as the positive control group. The hemolysis rate of the hydrogels was determined by the following equation: Hemolysis ratio (%) = (OD_s_- OD_n_)/(OD_p_ - OD_n_) × 100%, where OD_s_, OD_n_, OD_p_ were the absorbance of the experimental group, negative control group, and positive control group, respectively. The experiment was repeated three times.

**1.6 Antibacterial Activity and Zn^2+^ Release of Hydrogel.**

The antibacterial properties of the hydrogels were tested using E. coli (ATCC 25922) and S. aureus (ATCC 29213). The detailed experimental procedure was described in the experiments section in the Supporting Information. Zn^2+^ release from hydrogels was tested using an atomic absorption spectrophotometer (TAS-987, Beijing Purkinje General Instrument Co., Ltd., China). Briefly, 1 mL of PSA-ZnO hydrogel was immersed in 30 mL of simulated wound solution and incubated in an incubator at 100 rpm/min with the temperature set to 37 ℃. The release of Zn^2+^ at fixed time intervals was quantified using an atomic absorption spectrophotometer. The experiment was repeated three times. The antibacterial properties of the hydrogels were tested using E. coli and S. aureus. Briefly, the activated bacteria were prepared as bacterial suspensions and added into 96-well plates, and the OD value at wavelength 600 nm was measured by a microplate reader, and then the bacterial suspensions were diluted to the desired concentration according to the OD value. A 10 mm diameter hydrogel disc with 1mL of a certain concentration of bacterial suspension was added into the 24-well plate and incubated in a bacterial incubator at 37 ℃. After a total of 12 h incubation, 100 μL of bacterial suspension in the 24-well plate was sucked and applied to the solid medium, coated evenly, and the bacterial growth was observed after 24 h incubation. The number of bacterial CFU on the medium was calculated and photographed and recorded. The experiment was repeated three times.

**1.7 Antifouling Capability of Hydrogel.**

The antibacterial adsorption capacity of the hydrogels was assessed using *E. coli* and *S. aureus*. Briefly, the activated bacteria were prepared into bacterial suspensions and added into 96-well plates. The OD value at wavelength 600 nm was measured with a microplate reader, and then the bacterial suspensions were diluted to the desired concentration according to the OD value. Hydrogel discs with a diameter of 10mm and 1mL of a certain concentration of bacterial suspension were added to 24-well plates and incubated at 37 °C in a bacterial incubator. After a total of 6 h incubation, the hydrogel samples were removed and the surface of the hydrogel was washed three times with 1 mL PBS buffer solution. 100 μL of the third washing solution was absorbed and spread evenly in the solid medium, and the bacterial growth was observed and photographed after 24 h incubation in the incubator. The experiment was repeated three times.

To study the adsorption of fibroblasts on the hydrogel surface, hydrogel discs with a diameter of 10 mm and a thickness of 1 mm were made from dissolved and sterilized hydrogel samples, placed in triplicate in a 24-well plate, and incubated in a cell incubator with 5×10^4^ mL^-1^ of cell suspension. After 24 h of incubation, the medium was removed and the hydrogel discs were gently washed with PBS buffer solution. The cell staining solution was added to cover the surface of the hydrogel and incubated at 37 °C for 20 min. Finally, the cells were observed using an inverted fluorescence microscope and photographed and recorded.

The nonspecific protein adsorption assay was performed using a micro BCA protein assay kit with bovine serum albumin to determine the anti-protein adsorption capacity of the hydrogels. Dissolved and sterilized hydrogel samples were made into hydrogel discs with a diameter of 10mm and a thickness of 1 mm, placed in triplicate in a 24-well plate, and 1 mL of BSA protein solution with a concentration of 2 mg/mL was added. After incubation at 37 °C for 2 h, the BSA solution was removed and the hydrogel discs were rinsed with saline three times to remove the loosely adsorbed proteins on the surface. Then, the BSA firmly adsorbed on the hydrogels was separated after treatment with 1% sodium dodecyl sulfate (SDS) solution for 2 h. The concentration of adsorbed proteins was obtained by measuring the absorbance value at a wavelength of 570 nm using a microplate reader.

**Supplementary Figures**

**
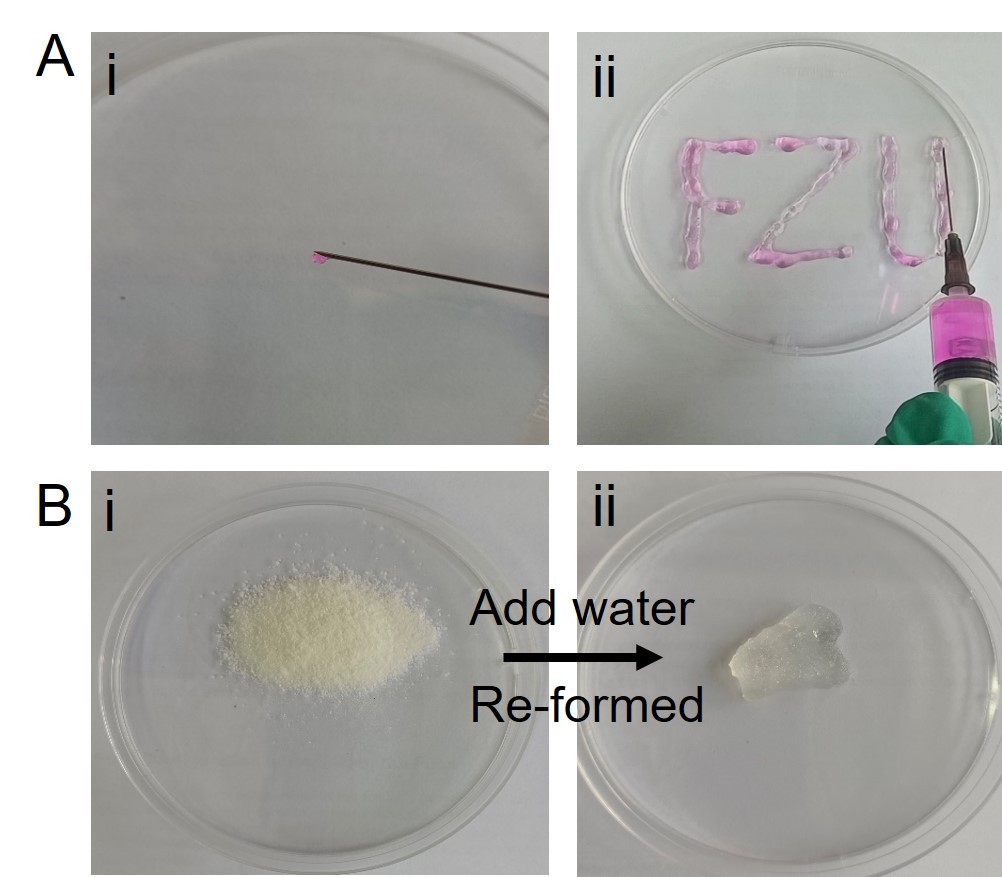
**

**Figure S1.** Photos of (A) injection properties and (B) hydrogel restoration of PSA-ZnO hydrogel.

**
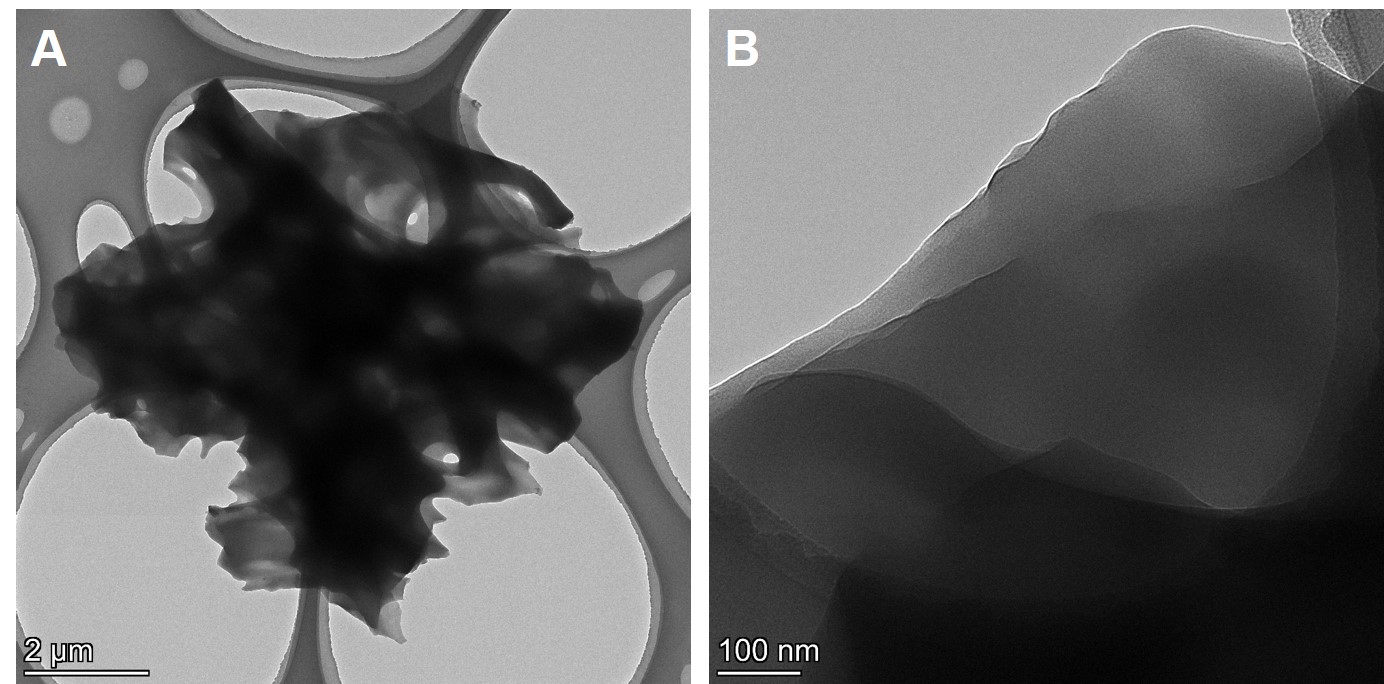
**

**Figure S2.** TEM of parent PSA hydrogel.


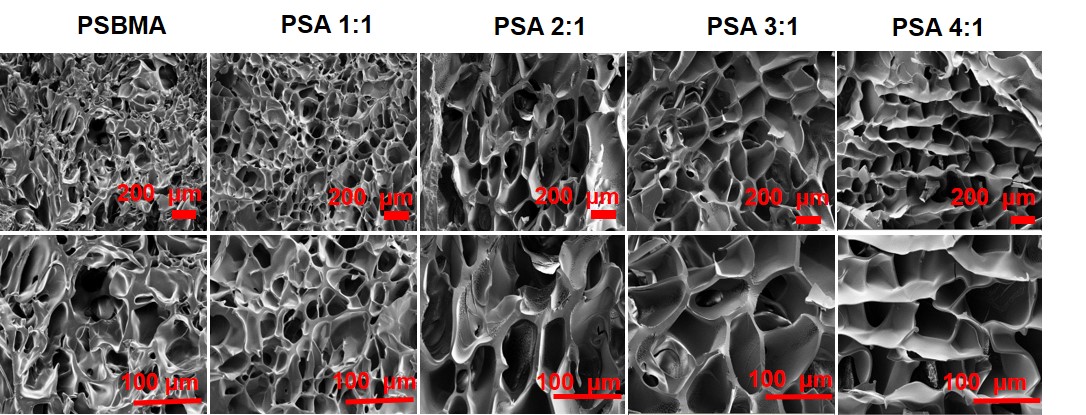


**Figure S3.** SEM of PSA hydrogel with different ratios of SMBA and A6ACA.

**
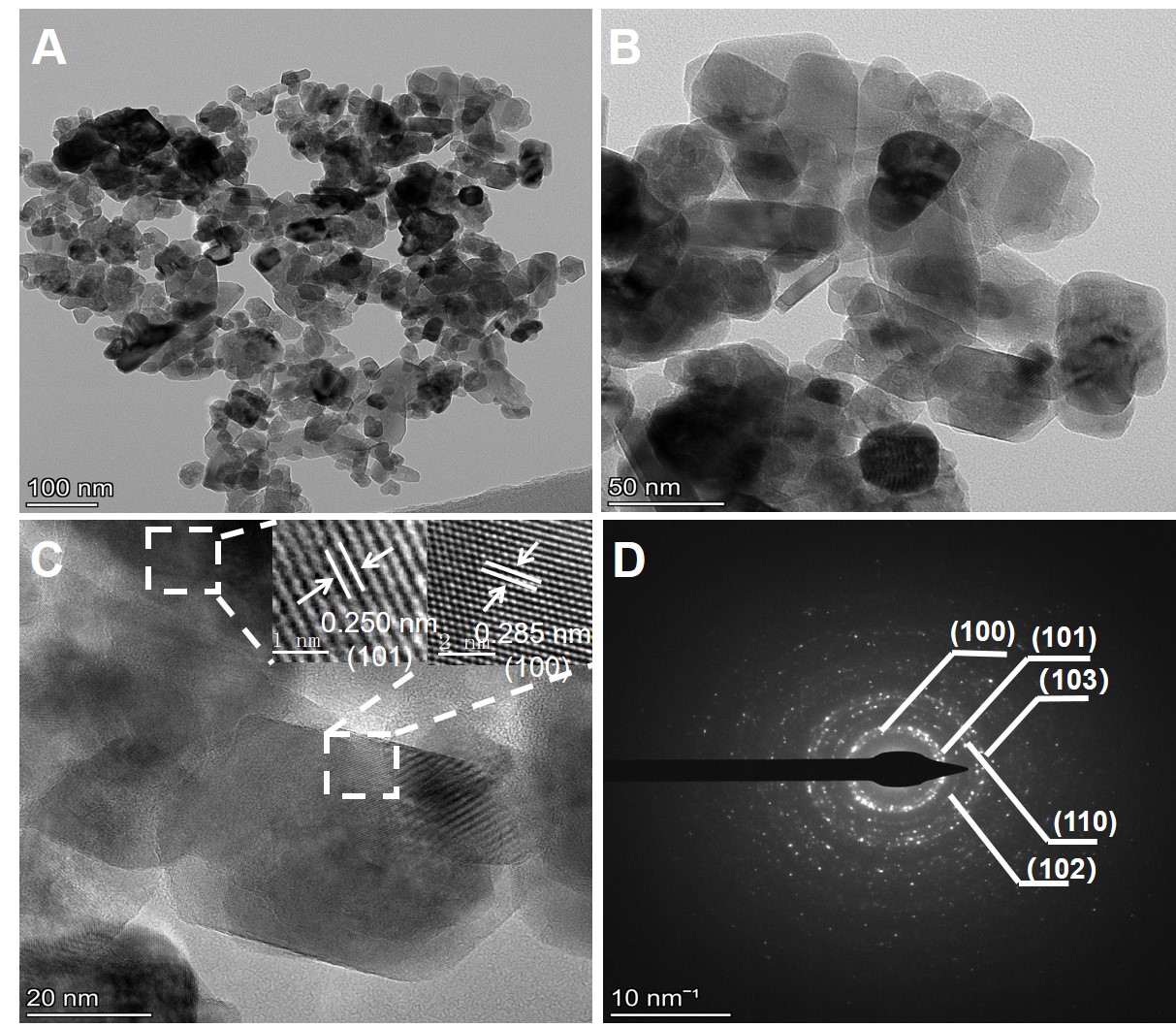
**

**Figure S4.** TEM of ZnO NPs.


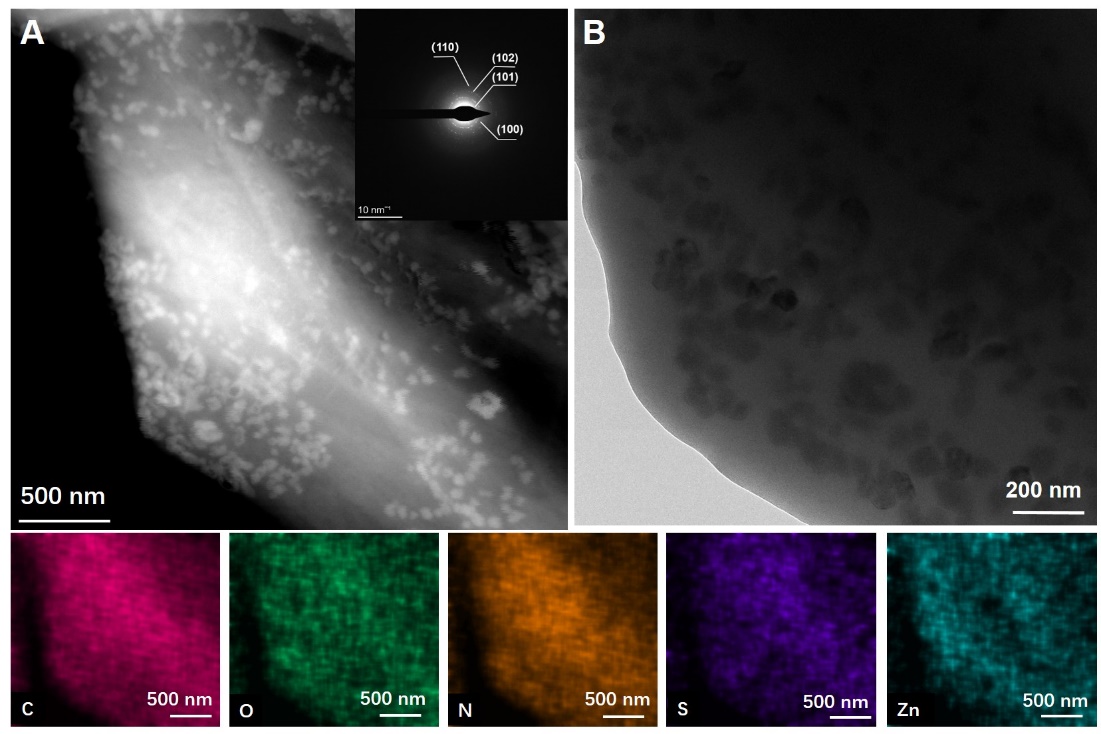


**Figure S5.** TEM image and elemental mapping images of C, O, N, S, and Zn ingredients of PSA-ZnO hydrogel.


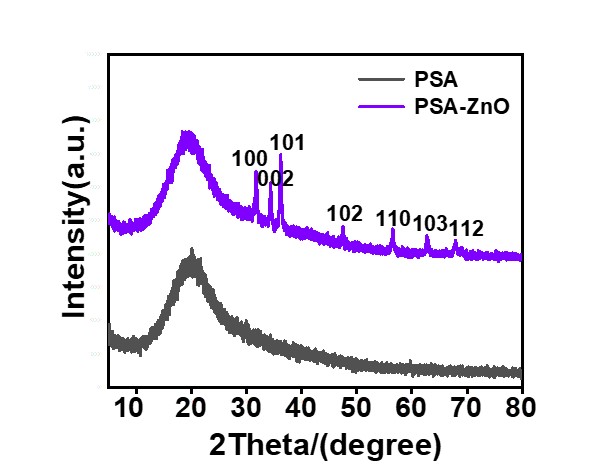


**Figure S6.** XRD patterns of PSA and PSA-ZnO hydrogels.

**
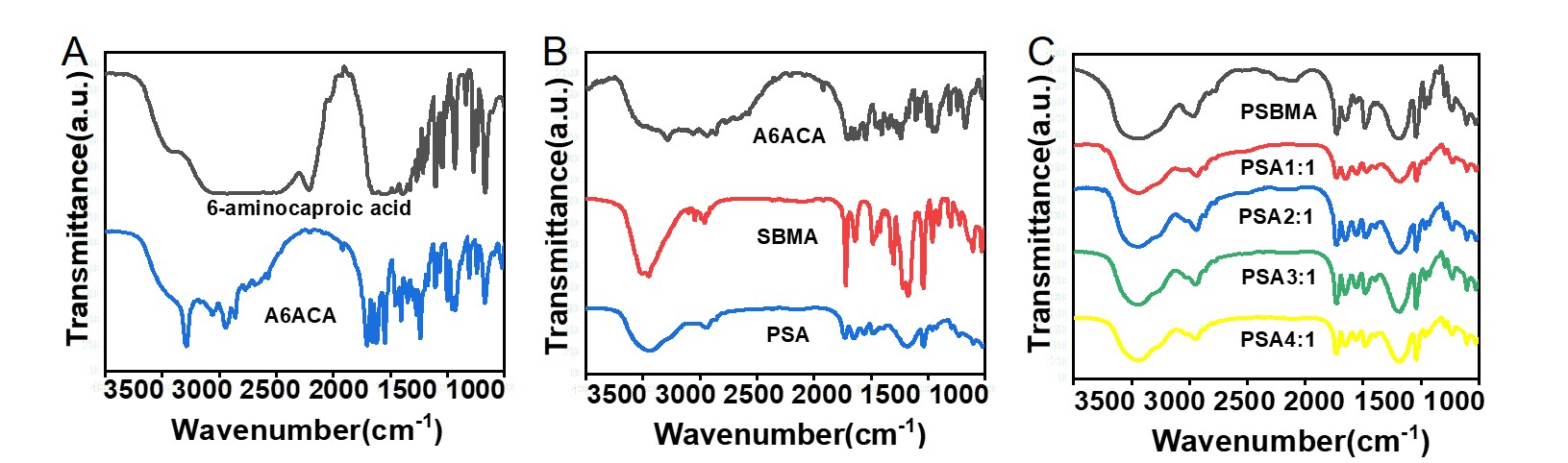
**

**Figure S7.** The FT-IR of (A) 6-aminocaproic acid andA6ACA, (B) monomer A6ACA, SBMA and PSA hydrogels, (C) PSA hydrogels with different ratios.


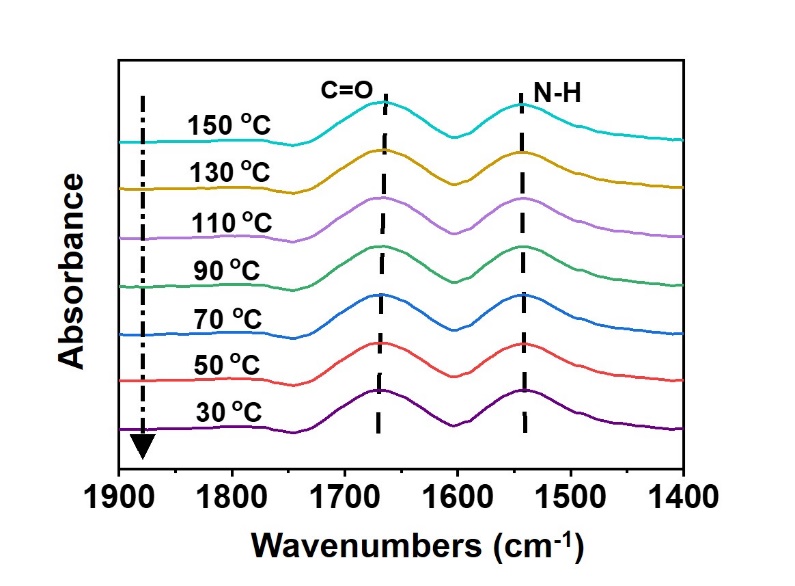


**Figure S8.** The cooling process of variable-temperature FTIR spectra of PSA hydrogel.


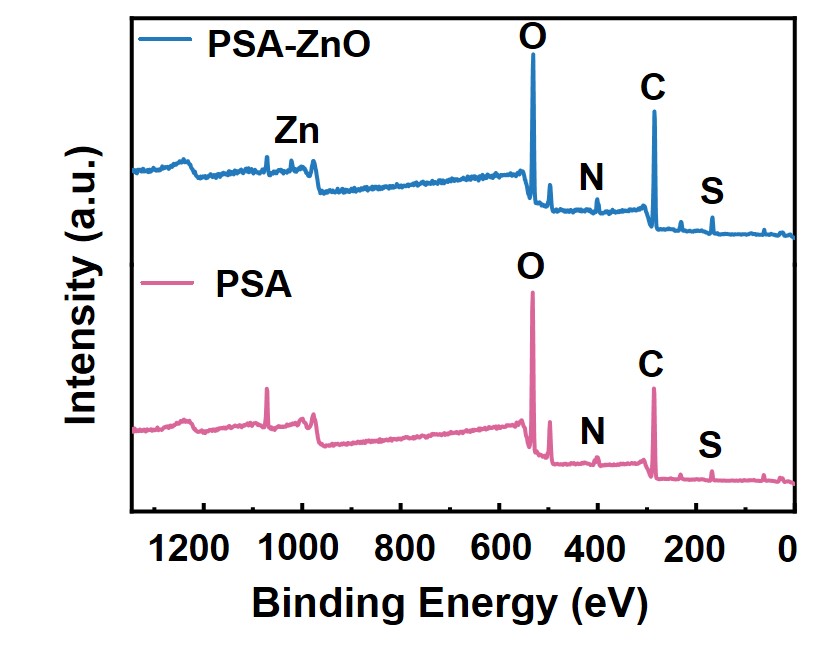


**Figure S9.** The complete XPS spectrum of parent PSA and PSA-ZnO hydrogel.

**
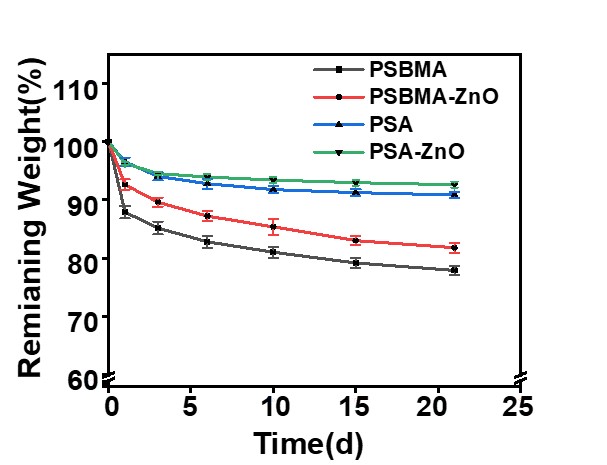
**

**Figure S10.** Degradation properties of hydrogels in PBS buffer solution at pH=7.4. Values are presented as mean ± SD. The “ns” means no significant difference. **p* < 0.05.

**

**

**Figure S11.** Controlled release of ZnO NPs in PSA-ZnO hydrogel.

**
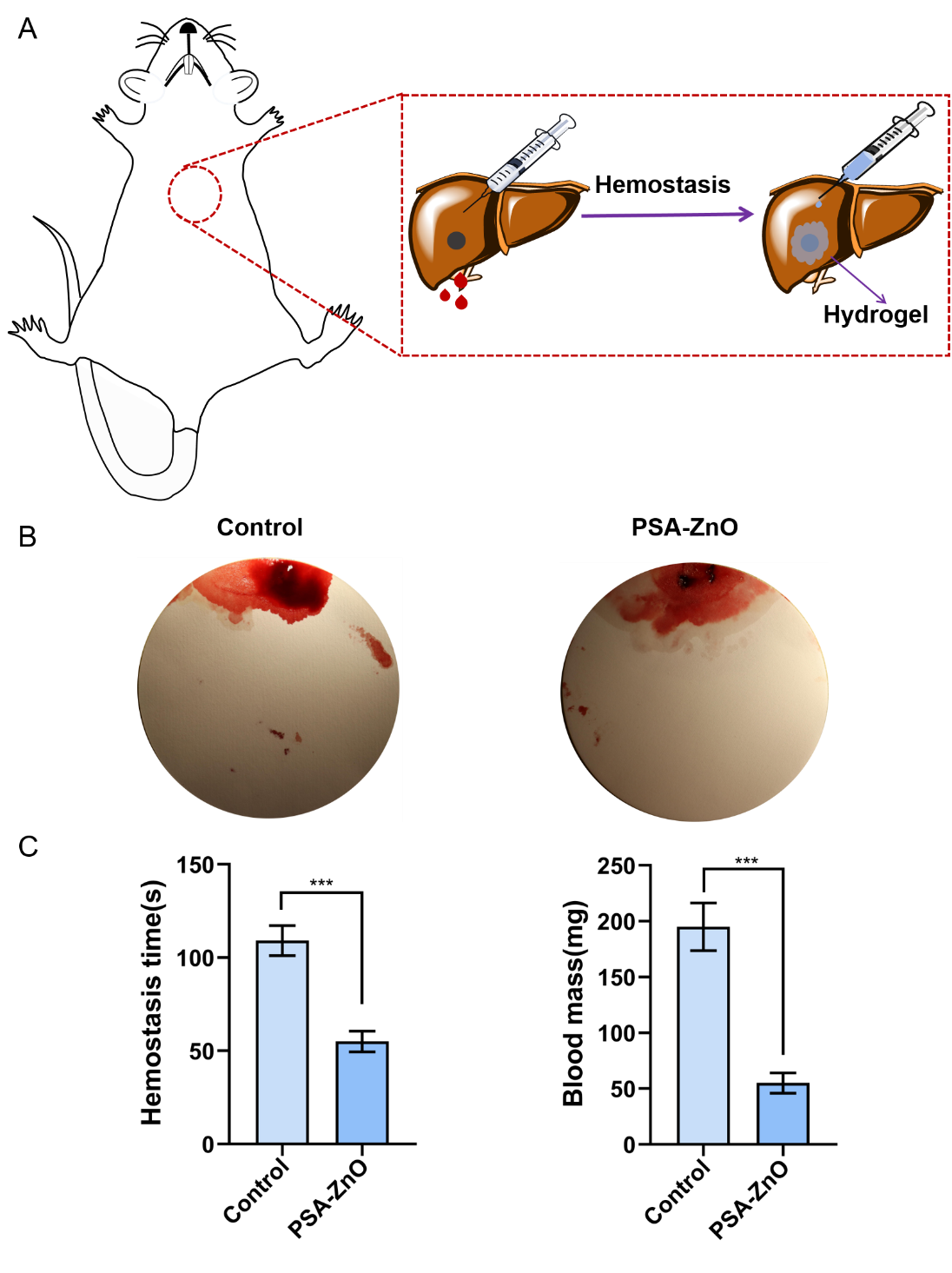
**

**Figure S12.** *In vivo* hemostatic properties of Hydrogel. (A) Achematic diagram of rat liver hemorrhage model. (B) Various treatment hemorrhage photos. (C) Hemostasis time and mass of the blood loss.

**
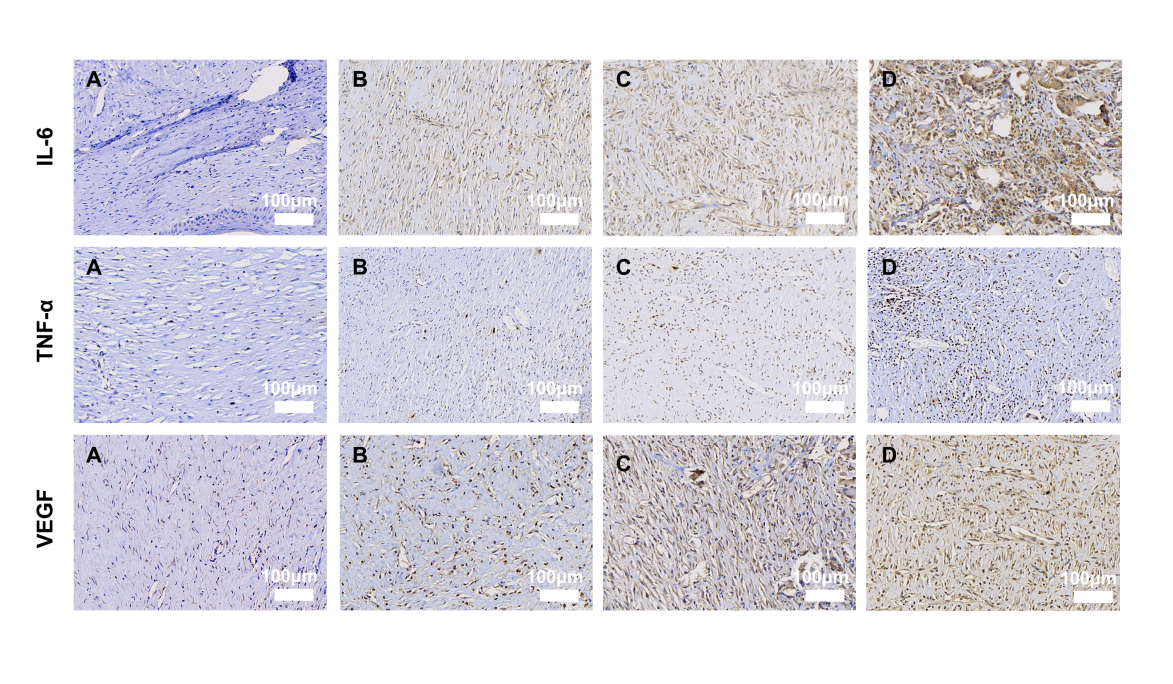
**

**Figure S13.** Immunohistochemical staining of IL-6, TNF-α, and VEGF expression in wound tissue. The staining intensity was classified as negative (A), weak (B), medium (C), and strong (D). Scale bar: 100 μm.

IL-6, TNF-α, and VEGF staining was localized to the cytoplasm, and positive results showed brownish-yellow, yellow granules. The stained sections were observed and judged by a pathologist under a microscope using a double-blind method, first scoring the staining intensity (0, negative; 1, weak; 2, moderate; 3, high) and then scoring the percentage of positive cells (0, 0% positive cells; 1, 1-10% positive cells; 2, 11-50% positive cells; 3, > 50% positive cells). The scores of the staining intensity score and the percentage of cells score were multiplied together to obtain the final score for each sample.

**
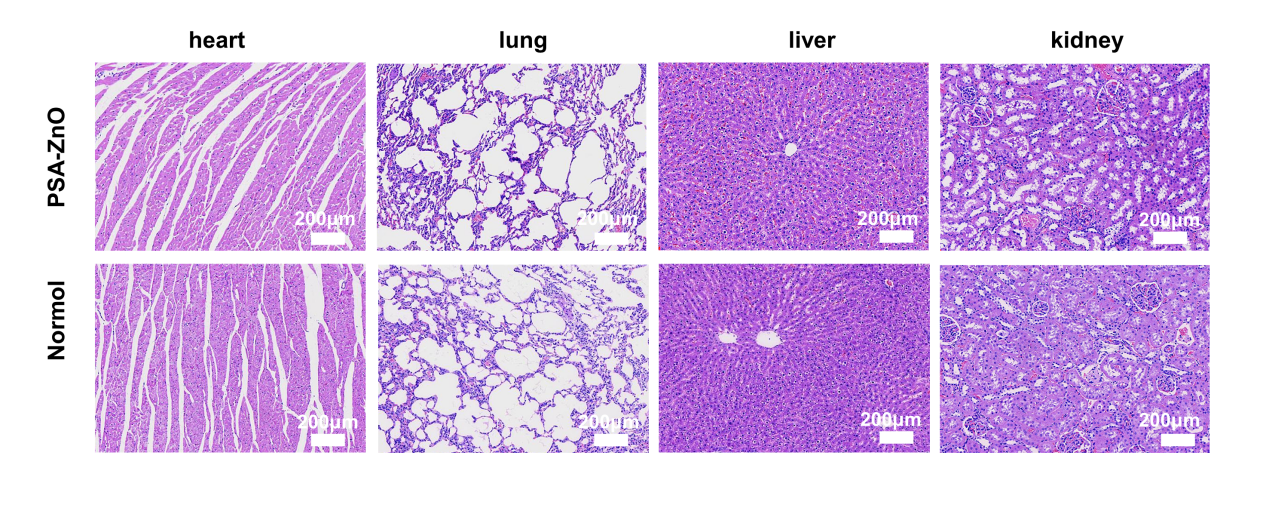
**

**Figure S14.** H&E staining of major organs of rats in PSA-ZnO hydrogel group. Scale bars: 200 μm.
